# Simultaneous Detection of EGFR and VEGF in Colorectal Cancer using Fluorescence-Raman Endoscopy

**DOI:** 10.1038/s41598-017-01020-y

**Published:** 2017-04-21

**Authors:** Yong-il Kim, Sinyoung Jeong, Kyung Oh Jung, Myung Geun Song, Chul-Hee Lee, Seock-jin Chung, Ji Yong Park, Myeong Geun Cha, Sung Gun Lee, Bong-Hyun Jun, Yun-Sang Lee, Do Won Hwang, Hyewon Youn, Keon Wook Kang, Yoon-Sik Lee, Dae Hong Jeong, Dong Soo Lee

**Affiliations:** 1 Department of Nuclear Medicine, Seoul National University College of Medicine, Seoul, Republic of Korea; 2 Department of Molecular Medicine and Biopharmaceutical Sciences, Graduate School of Convergence Science and Technology, Seoul National University, Seoul, Republic of Korea; 3 Department of Nuclear Medicine, CHA Bundang Medical Center, CHA University, Seongnam, Republic of Korea; 4 Department of Chemistry Education, Seoul National University, Seoul, Republic of Korea; 5 Wellman Center for Photomedicine, Harvard Medical School, Massachusetts General Hospital, Charlestown, Massachusetts USA; 6 Department of Biomedical Sciences, Seoul National University College of Medicine, Seoul, Republic of Korea; 7 Department of Bioscience and Biotechnology, Konkuk University, Seoul, Republic of Korea; 8 School of Chemical and Biological Engineering, Seoul National University, Seoul, Republic of Korea

## Abstract

Fluorescence endomicroscopy provides quick access to molecular targets, while Raman spectroscopy allows the detection of multiple molecular targets. Using a simultaneous fluorescence-Raman endoscopic system (FRES), we herein demonstrate its potential in cancer diagnosis in an orthotopically induced colorectal cancer (CRC) xenograft model. In the model, epidermal growth factor receptor (EGFR) and vascular endothelial growth factor (VEGF) were targeted with antibody-conjugated fluorescence and surface-enhanced Raman scattering (F-SERS) dots. FRES demonstrated fast signal detection and multiplex targeting ability using fluorescence and Raman signals to detect the F-SERS dots. In addition, FRES showed a multiplex targeting ability even on a subcentimeter-sized CRC after spraying with a dose of 50 µg F-SERS dots. In conclusion, molecular characteristics of tumor cells (EGFR in cancer cell membranes) and tumor microenvironments (VEGF in the extracellular matrix) could be simultaneously investigated when performing a colonoscopy.

## Introduction

Colonoscopy (standard white light endoscopy) is an essential tool for the localization and excision of suspected neoplastic lesions of colorectal cancer (CRC)^1, 2^. However, colonoscopy may result in misdiagnosis in up to 25% of cases, and polyps without malignant potential might be treated at high risk and cost to the patient^3^. Recent technological advancements in endoscopy procedures have improved the accuracy of endoscopic diagnosis of cancer^4^; examples include chromoendoscopy, light-scattering spectroscopy, autofluorescence imaging, endocystoscopy, high-resolution and magnifying endoscopy, etc^5^.

If applied to endoscopy, molecular imaging provides an opportunity to detect specific molecular targets of CRC early^6^. Fluorescence-based endomicroscopy (FBE) has been utilized to recognize these molecular targets in preclinical studies and is now used in clinical practice as a tool in image-guided cancer surgery^7^. FBE provides microscopic images using fluorescent dyes at the subcellular level although its use is limited to only one fluorescent dye at a time, which has limited the identification of potential multiple targets of a cancer^8^. Another technique, Raman spectroscopy, has also been introduced to discover the molecular characteristics of a cancer by distinguishing the inherent vibrational fingerprints of the cancer cells^9, 10^. Multiplex molecular imaging has been performed by utilizing the nanotags of surface-enhanced Raman scattering (SERS) with high sensitivity^11–14^, while its clinical applicability is under evaluation^5, 15^.

Previously, we adopted duplex fluorescence-SERS (F-SERS) probes against epidermal growth factor receptor (EGFR) and human epidermal growth factor receptor-2 (HER2) of breast cancer and combined FBE and Raman spectroscopy as one detection system called FRES (fluorescence-Raman endoscopic system), which successfully illustrated its value in subcutaneous tumor implants as a proof-of-concept^16^.

In orthotopic cancer implants, tumor cells are surrounded by various cells such as fibroblasts, immune and blood vessel cells, and also extracellular matrices. These are collectively called the tumor microenvironment in which its constitution is associated with the extent of tumor cell proliferation, angiogenesis, invasion, and patients’ survival; thus, each constituent should be examined and its role understood^17^. Hence, in this investigation, as a first step to simultaneously imaging a tumor and its microenvironment, we chose two targets for CRC: EGFR and vascular endothelial growth factor (VEGF)^18^. EGFR is targeted by cetuximab and VEGF by bevacizumab, both of which are used in clinical practice. Hence, when positive, the successful imaging of these two markers might guide their use to target the CRC of interest^19^.

**Figure 1:**
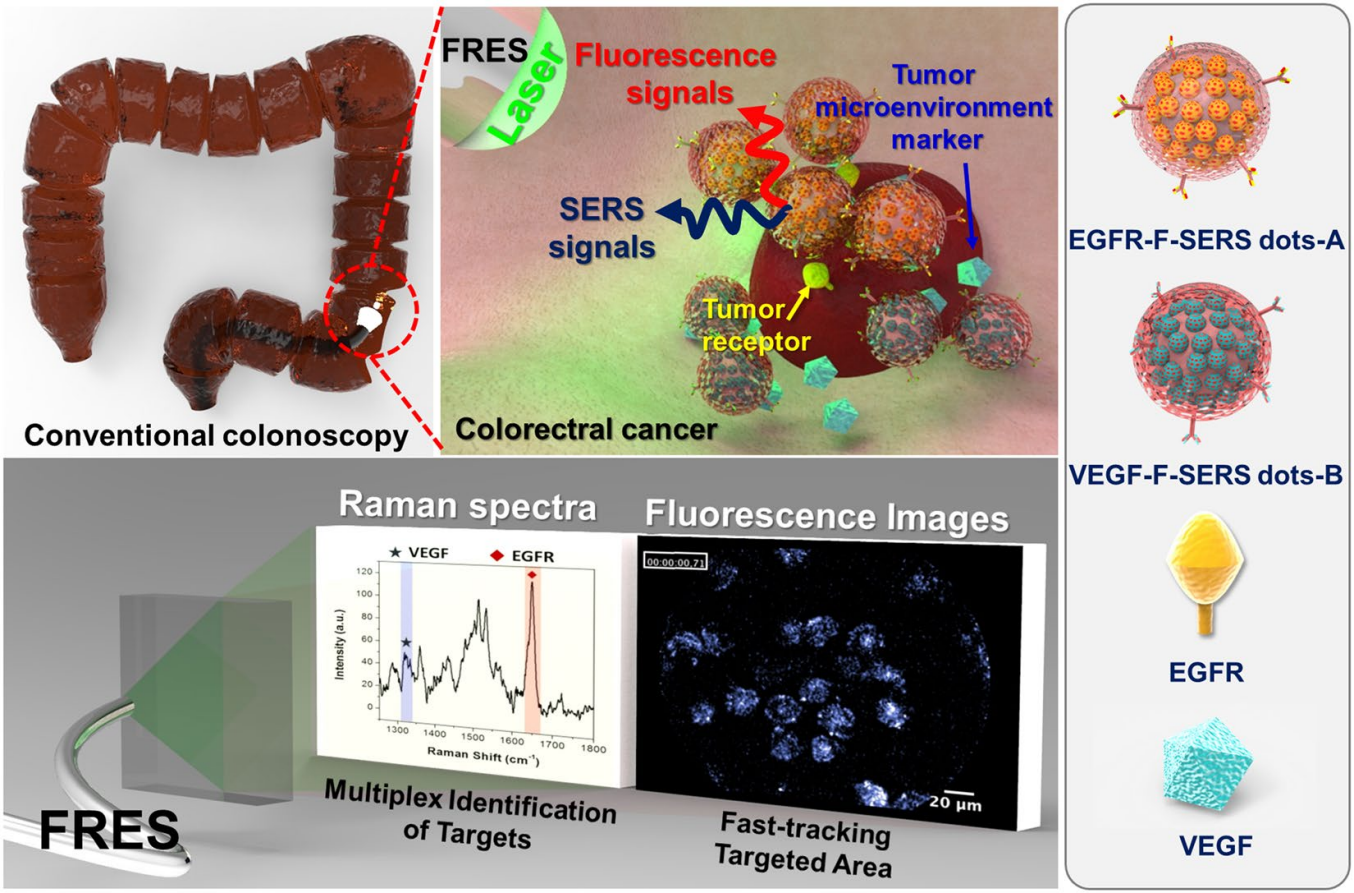
Schematic illustration of the *in vivo* multiplex molecular diagnosis on colorectal cancer using simultaneous fluorescence-Raman endoscopic system (FRES). FRES was able to detect fluorescence and Raman signals simultaneously for the molecular characterization of a tumor. When antibody-conjugated F-SERS dots were sprayed onto HT29-effluc colon cancer cells, the antibody-conjugated F-SERS dots bound to colon cancer cells [epidermal growth factor receptor (EGFR)] and tumor microenvironments [vascular endothelial growth factor (VEGF)]. FRES simultaneously utilizes the fluorescence signal of a fluorescent silica shell for fast signal detection [Alexa Fluor (AF) 610], and the Raman signals for multiplex targeting from the silver nanoparticles labeled by two kinds of Raman active compounds [rhodamine B isothiocyanate (RITC, -A) and fluorescein isothiocyanate (FITC, -B)].

In this investigation, we aimed to make FRES with F-SERS dots feasible in an orthotopic xenograft model of CRC (Fig. 1). Further to the validation of FRES/F-SERS endoscopy of EGFR/HER2, and once again in CRC, we validated the duplex targeting capability, the system’s detection limit (sensitivity) and reproducibility, and also its capacity for quantification and real-time imaging using F-SERS dots for EGFR (the target of cetuximab) and VEGF (the target of bevacizumab).

## Results

### Design of F-SERS dots and FRES

**Figure 2:**
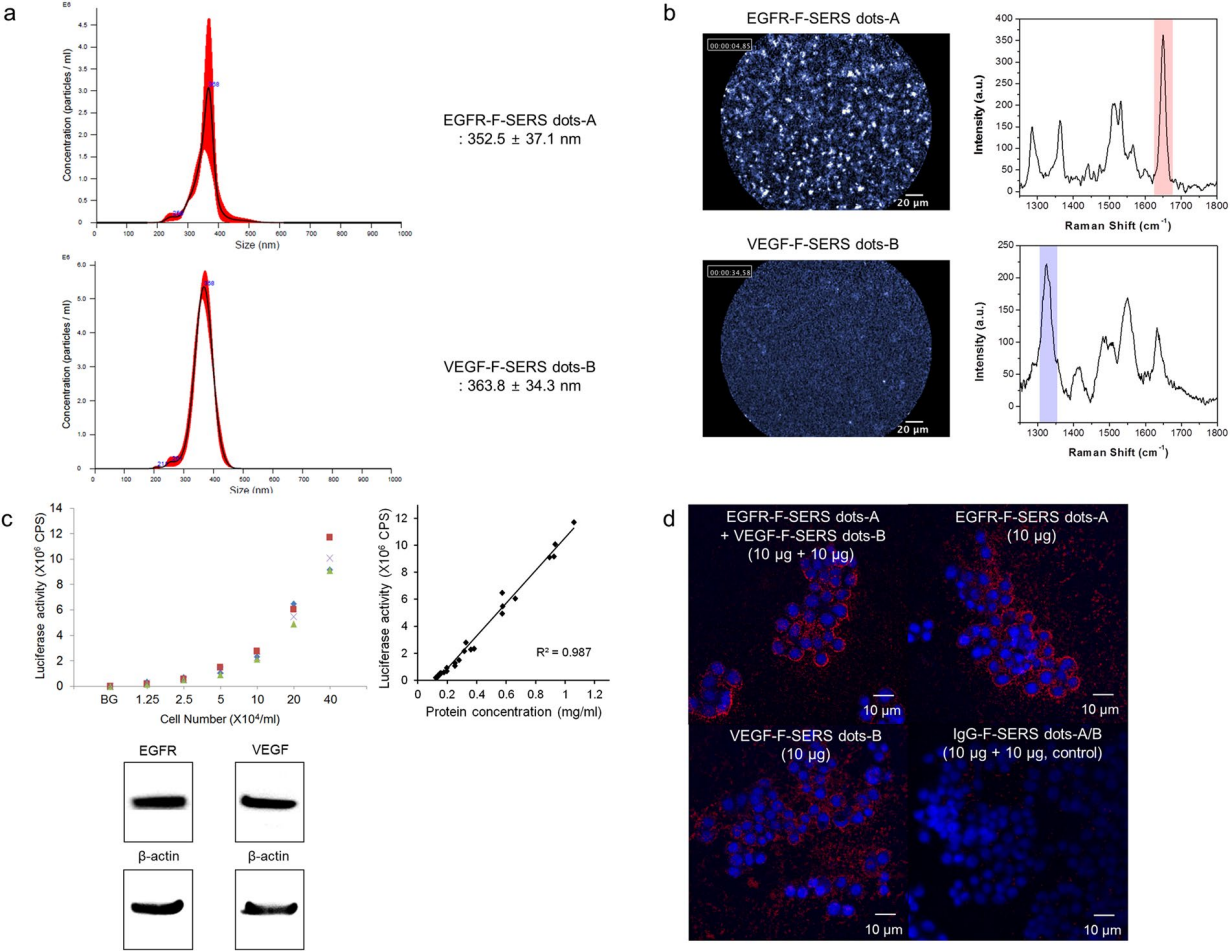
Characterization of fluorescence and surface enhanced Raman scattering nanoprobes (F-SERS dots), FRES, and colon cancer cell lines. (**a**) The size of the F-SERS and antibody-conjugated F-SERS dots. The size of the F-SERS-A and EGFR-F-SERS-A dots was 318.6  ± 20.1 nm and 352.5  ± 37.1 nm, respectively, and the size of the F-SERS-B and VEGF-F-SERS-B dots were 326.3  ± 25.9  nm and 363.8  ± 34.3  nm, respectively. (**b**) Evaluation of FRES using antibody-conjugated F-SERS dots. EGFR-F-SERS-A dots showed a small dotted fluorescence signal (AF 610) and a 1648 cm^−1^ intensity Raman signal (RITC).VEGF-F-SERS-B dots revealed small dotted fluorescence signals (AF 610) and a 1324 cm^−1^ intensity Raman signal (FITC) (a.u. = arbitrary unit). (**c**) Characterization of HT29-effluc colon cancer cells. HT29-effluc cells showed high luciferase activity according to seeded cell number and a significant positive correlation with protein concentration (R^2^ = 0.987, *P* < 0.001). HT29-effluc cells demonstrated EGFR and VEGF expression by western blot analysis (the loading control used was β-actin; CPS = count per sec). (**d**) Confocal laser scanning microscopy (CLSM) after spraying 10 µg of antibody-conjugated F-SERS dots onto cultured HT29-effluc cells. Multiple (EGFR-F-SERS-A and VEGF-F-SERS-B) or single (EGFR-F-SERS-A or VEGF-F-SERS-B) spraying of antibody-conjugated F-SERS dots were revealed by fluorescence, while the IgG-F-SERS-A/B dots (control) showed no fluorescence. Cell nuclei were labeled with DAPI.

F-SERS dots consist of silica spheres (*ca*. 180 nm) as a core, Raman active chemical-labeled silver nanoparticles (*ca*. 10 nm) generating SERS signals, and a fluorescent dye-conjugated silica shell emitting fluorescent signals, as shown in Supplementary Fig. 1. Thus, the F-SERS dots are able to simultaneously emit SERS and fluorescence signals from a single nanoparticle. Photostability was sustained at more than 90% fluorescence and more than 80% SERS signal for 300 sec continuous laser exposure (Supplementary Fig. 2). For multiplex detection, the F-SERS dots were labeled with a single fluorescent dye (AF610) and two Raman active molecules: rhodamine B isothiocyanate (RITC) and fluorescein isothiocyanate (FITC) for F-SERS-A and -B dots, respectively. F-SERS-A and F-SERS-B dots were conjugated with anti-EGFR and anti-VEGF antibodies, respectively, and their mean diameters were 352.5  ± 37.1 nm and 363.8  ± 34.3  nm, respectively (Fig. 2a and Supplementary Table 1). Fluorescence signals were represented as small bright dots, and Raman signals revealed the highest Raman band at 1648 cm^−1^ for RITC (EGFR-F-SERS-A dots) and at 1324 cm^−1^ for FITC (VEGF-F-SERS-B dots) (Fig. 2b). The limits for detection of fluorescence and SERS signals of F-SERS dots were ca. 0.5 pM and ca. 1 pM, respectively^16^.

### Characterization of the colon cancer cell line and FRES results *in vitro*

HT29-effluc colon cancer cells demonstrated gradually increased luciferase activity with seeded cell number and a significant positive correlation with protein concentration (R^2^ = 0.987, *P* < 0.001). Western blot analysis revealed EGFR and VEGF expression in this cancer cell line (Fig. 2c).

Antibody-conjugated F-SERS dots (EGFR-F-SERS-A and VEGF-F-SERS-B) were sprayed onto cultured HT29-effluc cells and binding was evaluated using confocal laser scanning microscopy (CLSM). When 10 µg of antibody-conjugated F-SERS dots were sprayed, fluorescence signals from them were observed. However, when 10 µg of IgG-F-SERS dots A/B were sprayed (control), fluorescence was not detected (Fig. 2d).

**Figure 3:**
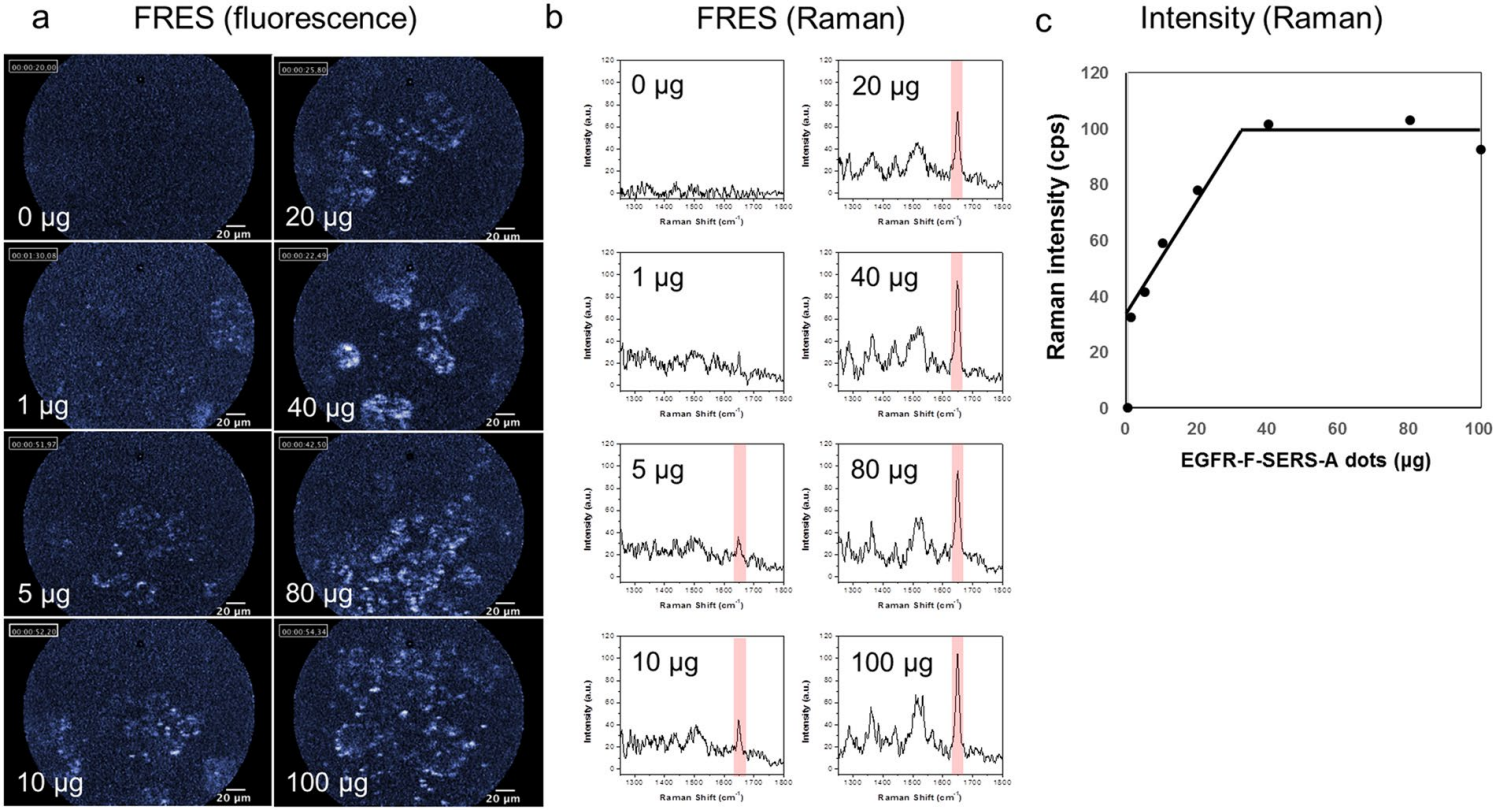
*In vitro* FRES result according to dose of EGFR-F-SERS-A dots. HT29-effluc cells (10^4^ cells/well) were seeded in an 8-well chambered coverglass with 300 μL of cell media per well. EGFR-F-SERS-A dots (0, 1, 5, 10, 20, 40, 80, and 100 μg) were added to the wells, incubated at room temperature for 10 min, and washed three times with phosphate-buffered saline (PBS), after which FRES imaging was performed. Using FRES, definite EGFR-F-SERS-A dots bound to tumor cells were found by (**a**) fluorescence and (**b**) Raman signals from 5 μg (10^4^/cm^2^). (**c**) When the Raman intensities at 1648 cm^−1^ were plotted, linear correlation was found until 40 μg, and saturation was found over 40 μg (10^4^/cm^2^).

The *in vitro* FRES study demonstrated that both fluorescence and Raman signals were detectable from 5 μg (10^4^ cells/cm^2^), and saturation of Raman intensity was observed at 40 μg (10^4^ cells/cm^2^); the FRES signal became distinct as the seeded cell density increased (Fig. 3 and Supplementary Fig. 3).

### Orthotopic CRC xenograft modeling after injection of 1 × 10^7^ HT29-effluc cells

**Figure 4:**
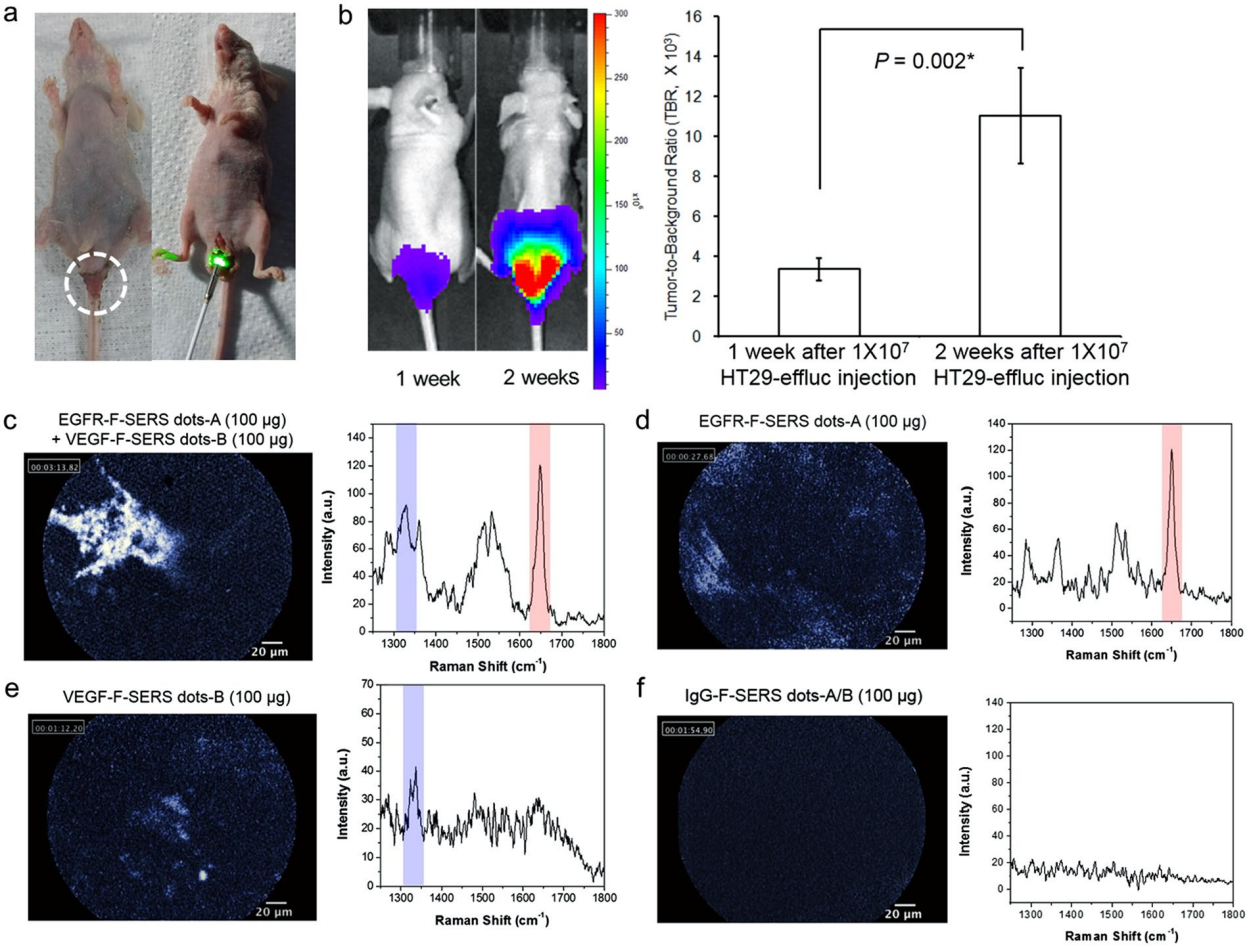
Multiplex targeting validation of FRES. (**a**) Orthotopic colorectal cancer (CRC) xenograft modeling for FRES. Two weeks after injecting 1 × 10^7^ HT29-effluc cells in mice, anal erosion was found (left) in most cases (86%, 12/14 mice), and the FRES imaging figure for the tumor-exposed system is shown on the right. (**b**) The bioluminescence image showed moderate-to-high activity in the colorectal area (left), and a significantly high tumor-to-background ratio (TBR, colorectal area/brain area) was identified at two weeks, compared with one week after injecting 1 × 10^7^ HT29-effluc cells (right, 11021 ± 2400 vs. 3354 ± 568, *P* = 0.002*; n = 14). (**c**–**f**) FRES for multiplex targeting ability validation. After 10 min of incubation and PBS washing, tumors were investigated using a FRES probe. (**c**) After spraying 100 µg of EGFR-F-SERS-A dots and 100 µg of VEGF-F-SERS-B dots, fluorescence signals were found by using AF 610, and two corresponding Raman signals were detected for RITC (-A) and FITC (-B) intensities. (**d**,**e**) After a single spraying dose of 100 µg each of EGFR-F-SERS-A and VEGF-F-SERS-B dots, fluorescence signals and corresponding single Raman signals [RITC (-A) and FITC (-B)] were found. (**f**) In contrast, spraying of IgG-F-SERS-A/B dots demonstrated no distinct fluorescence and Raman signals.

HT29-effluc orthotopic CRC xenograft model was established in BALB/c nude mice. From a total of 20 mice, 100% (20/20) survived one week after injection of 1 × 10^7^ HT29-effluc cells and 70% (14/20) survived after two weeks. Anal erosion symptoms (Fig. 4a) were found in 35% (7/20) of mice one week after injection and 86% (12/14) after two weeks (Supplementary Table 2). All mice showed moderate-to-high luciferase activity after one week (100%, 20/20). The tumor-to-background ratio (TBR) two weeks after injection was significantly higher compared with the TBR one week after injection, according to the bioluminescence images (3354 ± 568 vs. 11021 ± 2400, *P* = 0.002*; Fig. 4b). The largest mean diameter of colon cancer tumors was 15 mm (range: 12–19 mm).

### Validation of multiplex targeting ability of FRES

We attempted to validate the targeting ability of the two synthesized antibody-conjugated F-SERS dots. A total of 14 mice with tumors induced by injecting 1 × 10^7^ HT29-effluc cells after two weeks were studied: five in multiplex targeting, six in single targeting (three for EGFR and three for VEGF), and three as controls (IgG-F-SERS dots). FRES was able to simultaneously detect both fluorescence and multiple Raman spectra. The location of the targeted F-SERS dots was identified in real time using the fluorescence signals from the Alexa Fluor (AF) 610 dye. Raman bands at 1648 cm^−1^ and 1324 cm^−1^ were observed, which correspond to RITC for EGFR-F-SERS-A dots and FITC for VEGF-F-SERS-B dots, respectively (Fig. 4c). Definite signals were 80% (4/5) and probable signals were 20% (1/5) for mice in the multiplex targeting experiments. A single spray of antibody-conjugated F-SERS dots demonstrated definite fluorescence and corresponding Raman (RITC or FITC) signals in all cases (Fig. 4d and e). In contrast, in all cases of spraying IgG-F-SERS dots A/B as a control, no mice showed either fluorescence or Raman signals (Fig. 4f, Supplementary Fig. 4, and Supplementary Table 3). As a counter confirmation experiment, CLSM experiments were performed on the excised tumors which had shown definite binding of antibody-conjugated F-SERS dots to colon cancer cells but a lack of binding of IgG-F-SERS dots (Supplementary Fig. 5).

### Orthotopic CRC xenograft modeling after injection of 1 × 10^7^ HT29-effluc cells in a real-time endoscopic system

**Figure 5:**
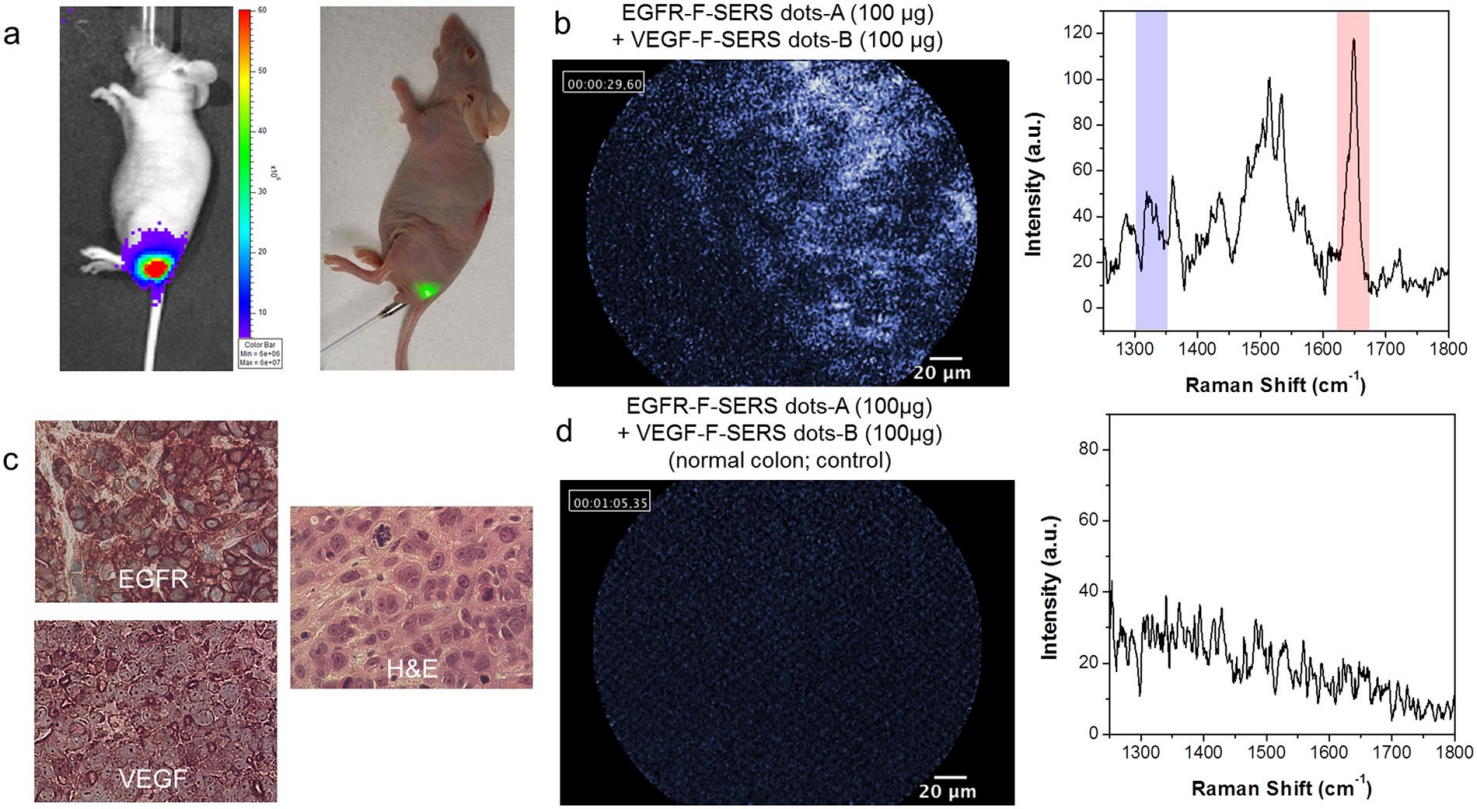
Evaluation of FRES in a real-time endoscopic system. (**a**) Orthotopic CRC xenograft modeling. One week after injecting 1 × 10^7^ HT29-effluc cells, bioluminescence imaging (left) showed moderate-to-high activity in the colorectal area of mice (lateral view; n = 20). The FRES imaging method used in a real-time endoscopic system is shown (right). (**b**) Mice with CRC (two weeks after injecting 1 × 10^7^ of HT29-effluc cells) were investigated in a real-time endoscopic study of FRES. After spraying 100 µg each of EGFR-F-SERS-A and VEGF-F-SERS-B dots, fluorescence signals were found as well as two corresponding Raman signals [RITC (-A) and FITC (-B)]. (**c**) Tumors were excised, fixed, and sectioned. EGFR and VEGF positivity was identified by immunohistochemistry (IHC), and tumor cell infiltration was observed by hematoxylin and eosin (H&E) staining. (**d**) After spraying 100 µg each of EGFR-F-SERS-A and VEGF-F-SERS-B dots on normal colons (control), no fluorescence or Raman signals were detected.

We next used the FRES imaging of the CRC xenograft model in a real-time endoscopic system. Among 20 studied mice with colorectal cancer, all showed moderate-to-high luciferase activity one week after injecting 1 × 10^7^ HT29-effluc cells (100%, 20/20) with the largest mean diameter of colon cancer being 13 mm (range: 11–17 mm); FRES imaging in the real-time endoscopic system is shown in Fig. 5a.

### Validation of FRES in a real-time endoscopic system

We validated FRES imaging as a tool to detect tumors during real-time endoscopy. A total of 20 mice were investigated in a real-time endoscopic study of FRES (100 µg EGFR-F-SERS-A dots + 100 µg VEGF-F-SERS-B dots). In addition, 9 mice were studied as controls for a normal colon (100 µg EGFR-F-SERS-A dots + 100 µg VEGF-F-SERS-B dots). FRES showed specific targeting ability in a multiplex capacity in all the cases. Definite signals were observed in 75% (15/20) and probable signals in 25% (5/20) of mice with colorectal cancer (Fig. 5b). Immunohistochemistry (IHC) results demonstrated both EGFR and VEGF positivity in excised colorectal cancer, and hematoxylin and eosin (H&E) staining revealed tumor cell infiltration (Fig. 5c). Fluorescence and Raman signals were not observed in the normal colon controls (Fig. 5d, Supplementary Fig. 5, and Supplementary Table 4).

### Orthotopic CRC xenograft modeling after injection of 5 × 10^6^ HT29-effluc cells in a tumor-exposed system

**Figure 6:**
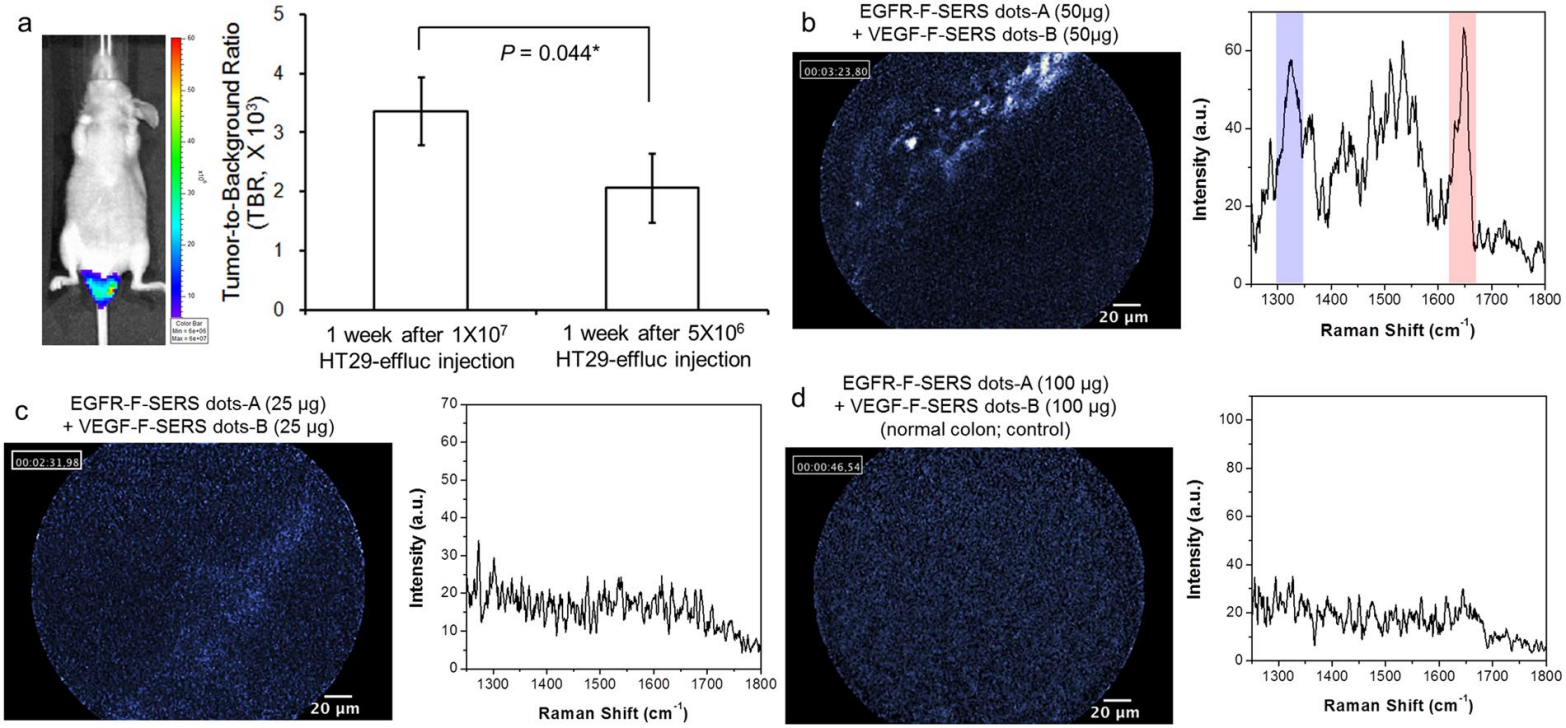
Evaluation of sensitivity and lower dose limit of FRES. (**a**) Orthotopic CRC xenograft modeling for small tumors. After one week of injecting 5 × 10^6^ HT29-effluc cells in mice, bioluminescence imaging showed mild-to-moderate activity in the colorectal area (left). A significantly lower TBR was found compared with that of mice one week after injecting 1 × 10^7^ HT29-effluc cells (2,055 ± 578 vs. 3,354 ± 568, *P* = 0.044*) (right). (**b**) After spraying 50 µg of EGFR-F-SERS-A and VEGF-F-SERS-B dots onto small tumors in a CRC xenograft model, fluorescence signals were observed, and two corresponding Raman signals [RITC (-A) and FITC (-B)] were found. (**c**) On the other hand, after spraying 25 µg each of EGFR-F-SERS-A and VEGF-F-SERS-B dots, a faint fluorescence signal and no Raman signal was found in some cases (20%, 2/10). (**d**) In the normal colon (control), no fluorescence or Raman signals were found corresponding EGFR-F-SERS-A and VEGF-F-SERS-B dots.

The establishment of a xenograft model with small tumors using a lower dose of HT29-effuc cells was attempted. Among the 50 mice in the study group with tumors induced by 5 × 10^6^ HT29-effluc cells injection after one week, all showed mild-to-moderate luciferase activity (100%, 50/50), and the TBR showed significantly lower activity compared with the TBR of the group one week after injecting 1 × 10^7^ HT29-effluc cells (2055 ± 578 vs. 3354 ± 568, *P* = 0.044*; Fig. 6a). The largest mean diameter of colon cancer tumors was 7 m (range: 5–9 mm).

### Identification of FRES sensitivity and the lower dose limit of antibody-conjugated F-SERS dots in small tumors

Twenty mice with colon cancer were treated with high doses (100 µg EGFR-F-SERS-A dots + 100 µg VEGF-F-SERS-B), another twenty with medium doses (50 µg EGFR-F-SERS-A dots + 50 µg VEGF-F-SERS-B), and the other ten with low doses (25 µg EGFR-F-SERS-A dots + 25 µg VEGF-F-SERS-B) of antibody-conjugated F-SERS dots for the determination of the sensitivity and the identification of the lower dose limit for FRES. In addition, eight mice as controls with normal colons were treated with high doses (100 µg EGFR-F-SERS-A dots + 100 µg VEGF-F-SERS-B dots). FRES was able to detect CRC at high and medium doses of antibody-conjugated F-SERS dots in all of the cases. Definite signals were detected in 80% (16/20) and probable signals in 20% (4/20) of animals for high dose antibody-conjugated F-SERS dots, and definite signals were detected in 75% (15/20) and probable signals in 25% (5/20) for medium dose antibody-conjugated F-SERS dots (Fig. 6b). For the low dose antibody-conjugated F-SERS dots group, 50% (5/10) showed definite signals and 30% (3/10) were probable signals. No signal was found for low dose antibody-conjugated F-SERS dots in 20% (2/10) (Fig. 6c). Fluorescence and Raman signals were not observed in all of the control group of normal colon cases (Fig. 6d, Supplementary Fig. 7, and Supplementary Table 5). CLSM images of tumor sections showed definite binding when high, medium, and low doses of antibody-conjugated F-SERS dots were used, while binding of antibody-conjugated F-SERS dots on normal colons was not detected (Supplementary Fig. 8).

## Discussion

We have shown that FRES is a useful endoscopic tool in an orthotopic CRC xenograft model using antibody-conjugated F-SERS dots. FRES has advantages in that it allows the fast simultaneous detection of fluorescence and Raman signals. Fluorescence signals were useful for fast tracking of lesions, and Raman signals were helpful for multiplex target identification (both tumor cells and tumor microenvironment) and quantification. Most of all, we identified that FRES could be useful in an *in vivo* real-time endoscopic system as a practical diagnostic tool. In addition, we confirmed the multiplex targeting ability of FRES in subcentimeter-sized colorectal cancers by lowering the spraying dose of antibody-conjugated F-SERS dots according to tumor size, thereby verifying the sensitivity of FRES.

The main advantage of FRES is that it can be easily applied to a conventional endoscopic system since it has an optical fiber bundle probe which can be inserted into the accessory channel of a conventional endoscope^20^. Fluorescence-based techniques can improve the detection ability of a routine colonoscopy with low cost and the use of a probe that lacks toxicity, but they also have limitations in that usually, only one target signal can be detected due to its broad bandwidth and autofluorescence from tissue leads to false-positive results^21–23^. In contrast, SERS-based techniques have advantages such as high sensitivity and multiplex targeting abilities, but show limitations such as a small field of view and limited study data^23–26^. In contrast, FRES can take advantage of combining a large field of view (fluorescence signal) and a multiplex ability (SERS signal) for dual-modality imaging, resulting in the rapid and accurate characterization of lesions.

The molecular characterization of CRC is very important for the selection of the most appropriate treatment option as well as for patients’ prognosis and quality of life. For CRC, EGFR (anti-EGFR monoclonal antibody: cetuximab) and VEGF (anti-VEGF monoclonal antibody: bevacizumab) targeting is the standard therapy that prolongs survival in patients with metastatic disease^19^, and previous studies have shown that molecular imaging of EGFR^27^ and VEGF^28, 29^ can be performed *in vivo*, so we decided that the FRES imaging targets should be EGFR and VEGF. As EGFR targets the cellular membrane^30^, and VEGF targets the extracellular matrix^31^, our study showed simultaneous tumor cell and tumor microenvironment imaging. In addition, our previous pilot study for targeting EGFR and HER2 using the HT29-effluc cells xenograft model showed duplex cellular targeting ability (Supplementary Fig. 9).

IHC has been routinely used for molecular diagnosis but can yield false positive/negative results due to technical and interpretative pitfalls^32^. Fixation and tissue processing artifacts, as well as antigen retrieval errors during IHC procedures, can hamper the exact evaluation of a tumor’s characteristics. Additionally, 2–3 days delay in evaluation after a colonoscopy may cause a loss of optimal treatment time^33, 34^. With FRES, we can achieve multiple molecular characteristics while performing real-time colonoscopies with high accuracy, resulting in rapid and effective diagnoses.

For the successful clinical application of FRES, major issues such as stability, signal sensitivity, specificity, and toxicity of F-SERS dots were properly addressed^35^. First, the optical stability of the F-SERS dots was tested. Fluorescence and Raman signals did not decline or change during surface modification and antibody conjugation, and emitted the same fluorescence and Raman signal intensity more than 1 year after synthesis. In regard to the issue of sensitivity, we performed a FRES study on small tumors (less than 1 cm) that showed a similar multiplex targeting ability to the one for large tumors (greater than 1 cm). For the specificity issue, we performed a blocking study and demonstrated that FRES signals were selectively blocked by cold antibodies (Supplementary Fig. 10). In regard to toxicity, we reduced systemic toxicity by using a topical administration method via directly spraying antibody-conjugated F-SERS dots onto the tumor, thereby bypassing the major disadvantages of intravenous injection: systemic toxicity caused by accumulation in major organs (lung, liver and spleen)^36–39^ and a decreased tumor-targeting ability due to the large size of antibody-conjugated F-SERS dots^40^. In addition, we introduced a silica shell (nontoxic coating) for the F-SERS dots, which is well known to reduce the toxicity of nanoparticles^41–43^. Furthermore, we observed that antibody-conjugated F-SERS dots did not accumulate in the normal colon after washing, thus further reducing any toxicity towards normal organs. Last, a half dose of antibody-conjugated F-SERS dots used for small tumors showed similar FRES results to a high dose; spraying 50 + 50 µg of antibody-conjugated F-SERS dots (5 nmol) onto a small tumor was found to be acceptable.

One important point of our study is the quantification of Raman intensity. As the Raman signal showed high signal intensity, the intensity could be quantified by measuring the highest peak of the Raman bands. The *in vitro* study demonstrated that Raman intensity increases according to EGFR-F-SERS-A dot doses, or seeded cell density. Moreover, we were able to evaluate different molecular expression within tumors using FRES with quantification of Raman intensity *ex vivo* (Supplementary Fig. 11)^44^.

In this study, we achieved duplex targeting using both types of F-SERS dots although we did not fully exploit the SERS- capability in multiplex testing. Furthermore, as we targeted the EGFR tumor marker on the surface of tumor cells and tumor-secreted VEGF in the tumor microenvironment, this study could now be extended to include the surface epitopes of immune or stromal cells including fibroblasts and other secreted peptides or cytokines prevalent in the tumor microenvironment. Though surface modification of F-SERS dots with three commercialized monoclonal antibodies (cetuximab, bevacizumab, and herceptin) was successful, surface labeling with other collections of antibodies should be performed and validated individually, and for optimal multiplexing, the selection of the SERS dots as well as their preparation and validation should be performed once again.

In conclusion, we demonstrated that FRES can be utilized in multiplex molecular diagnosis which can be easily applied to routine endoscopy. By using this technique, the multiple molecular characteristics of a tumor (tumor cell and tumor microenvironment) can be acquired simultaneously while performing a colonoscopy, and this could be effective in the early and most appropriate selection of treatment options. In addition, FRES can be applied to small tumors by lowering the dose of topically sprayed F-SERS dots, which can minimize the toxicity problem when applied clinically. We expect that FRES will eventually become an appropriate endoscopic technique for the rapid and specific diagnosis of CRC.

## Methods

### Fluorescence-Raman endoscopic system (FRES)

The optical setup for FRES is described in our previous study^16^. In brief, in order to simultaneously detect both fluorescence and Raman signals, FRES consists of three units: (1) a dual-axis laser-scanning unit, (2) a separation unit, and (3) a signal-detecting unit. The dual-axis laser-scanning unit (Raman prototype of Cell-Vizio^TM^; Mauna Kea Technologies, Paris, France) has two orthogonally oscillating mirrors that allow the sequential injection of a laser line into each one of a bundle of optical fibers for light incidence. To introduce the laser line to the sample and collect optical signals from the sample, a fiber-optic probe having a 2.6 mm external diameter, a 60 μm working distance, and a 240 μm field-of-view was utilized. As an excitation source, a continuous wave diode-pumped solid-state 532 nm laser (Cobolt Samba^TM^; Cobolt AB, Solna, Sweden) was used, coupled with a single mode fiber. The separation unit uses two optical filters: a long-pass dichroic filter (FF593-Di03-25D; Semrock Inc., Rochester, NY, USA) for passing the fluorescence signal from the collected signal, and an edge filter (LP03-532RS-25; Semrock Inc., Rochester, NY, USA) for the rejection of Rayleigh-lights and the passing of Raman-scattering lights. To simultaneously provide real-time fluorescence images and Raman spectra, the signal detecting unit consists of two independent detectors: an avalanche photodiode for fluorescence signal detection and a spectrometer (SR 303i-A; Andor Technology, Belfast, UK) with a thermo-electrically cooled CCD detector (DV401A-BV; Andor Technology, Belfast, UK) for Raman signal detection. The collected fluorescence signals were processed into real-time fluorescence images by imaging software (ImageCell; Mauna Kea Technologies, Paris, France) (Supplementary Fig. 12).

### Preparation of fluorescence & surface-enhanced Raman scattering probes (F-SERS dots)

F-SERS dots were synthesized according to the previously reported method^16^. Briefly, 200 nm-sized silica nanoparticles (NPs) were synthesized according to the Stöber method^45^. Tetraethyl orthosilicate (TEOS; 1.6 mL) in 40 mL of absolute ethanol was reacted with 3 mL ammonium hydroxide (NH_4_OH) for 20 h at room temperature. After thiol-functionalization on the surface of silica NPs (10 mg) by reacting with 100 μL 3-mercaptopropyltrimethoxysilane (MPTS) and 20 μL NH_4_OH (27%) for 2 h at room temperature, silver NPs were directly introduced onto the surface of the silica NPs by reducing 3 mM silver nitrate (AgNO_3_) in ethylene glycol with 10 mM octylamine for 1 h. To encode with Raman-labeling compounds, silver-embedded silica (Ag-Si) NPs were treated with 1 mM RITC or 1 mM FITC, respectively. The Raman-labeled Ag-Si NPs were then encapsulated with a silica shell using TEOS and NH_4_OH. Further encapsulation with fluorescent dye (AF 610)-conjugated silica shells was performed in a TEOS (10 μL), NH_4_OH (0.5 mL, 27%), and AF610-3-aminopropyltriethoxysilane (APTES; 55 μL) conjugated solution previously prepared by reacting 5 μL AF610 (8 mM in dimethyl sulfoxide (DMSO)) with 50 μL APTES (19.2 mM in ethanol) for 15 h. The final synthesized nanoprobes [F-SERS-A dots (using RITC) and -B dots (using FITC)] were washed with ethanol several times for purification purposes and then redispersed in ethanol.

### Establishment of colorectal cancer cell lines and their evaluation

The human colorectal adenocarcinoma cell line (HT29), which is known to express EGFR and VEGF^46^, was acquired from the Korean Cell Line Bank (KCLB, Seoul, Korea). HT29 cells were cultured in Dulbecco’s Modified Eagle’s Medium (DMEM; Invitrogen, Grand Island, NY, USA) supplemented with 10% fetal bovine serum, 100 U/mL penicillin, and 100 g/mL streptomycin at 37 °C in a humidified atmosphere containing 5% CO_2_. A retroviral vector containing codon-optimized firefly luciferase complementary DNA (effluc), and Thy1.1 (CD90.1), which is linked with an internal ribosomal entry site (IRES), was constructed for use in enhanced bioluminescence cell imaging^47, 48^. HT29 cells were transfected with a recombinant retroviral vector encoding a luciferase gene (HT29-effluc). To determine whether the luciferase gene was expressed in the established HT29-effluc cells, the latter were seeded into a 24-well plate and an *in vitro* luciferase assay was performed according to the seeded cell number and protein concentration using a luminometer (TR717p; Applied Biosystems, Grand Island, NY, USA). Protein concentrations were measured using BCA protein assay kits (Thermo Scientific, Rockford, IL, USA).

### Modeling of orthotopic colorectal cancer (CRC) xenografts

All animal protocols were approved by the Institutional Animal Care and Use Committee of the Seoul National University College of Medicine, and all experiments were performed in accordance with relevant guidelines and regulations. Six-week-old male BALB/c nude mice were obtained from Orient Bio, Inc. (Seoul, Korea) and housed in a specific, pathogen-free environment. CRC xenograft mice were divided into three groups:

1. Injection of 1 × 10^7^ of HT29-effluc cells for FRES imaging in a tumor-exposed system (n = 20; validation of the multiplex targeting ability of FRES).
2. Injection of 1 × 10^7^ of HT29-effluc cells for FRES imaging in an endoscopic system (n = 20; validation of FRES as a real-time endoscopic system).
3. Injection of 5 × 10^6^ of HT29-effluc cells for FRES imaging in a tumor-exposed system (n = 50; validation of FRES sensitivity and the lower dose limit of antibody-conjugated F-SERS dots for small tumors).

Before each injection of tumor cells, mice were anesthetized with an intramuscular injection of 200 µL of 0.5% zoletil 50 (tiletamine-zolazepam; Virbac S.A., Carros, France) and 0.2% xylazine (Rompun; Bayer, Leverkusen, Germany) solution (1:1). Using a 30-gauge needle, a 0.1 mL volume of complex (containing tumor cells at a 1:1 ratio in medium/matrigel) was injected into the posterior colorectal wall via the anus^49, 50^. Tumor growth was investigated over two weeks for the injection of 1 × 10^7^of HT29-effluc cells groups, and over one week for the injection of 5 × 10^6^ of HT29-effluc cells group.

### *In vitro* and *in vivo* validation of FRES measurements

As a tumor targeting agent, antibody-conjugated F-SERS dots were measured in a conical tube. Fluorescence and Raman signals were identified simultaneously to investigate the binding of antibody-conjugated F-SERS dots with colon cancer cells. First, we identified the sensitivity and correlation of Raman intensity according to dose of antibody-conjugated F-SERS dots using FRES. HT29-effluc cells (10^4^ cells/well) were seeded in an 8-well chambered coverglass (Lab-Tek; Thermo Scientific, Rochester, NY, USA) with 300 μL of cell media per well. After 24 h incubation at 37 °C, cells were fixed with 4% paraformaldehyde (Wako, Osaka, Japan) for 20 min, and washed three times with phosphate-buffered saline (PBS). EGFR-F-SERS-A dots (0, 1, 5, 10, 20, 40, 80 and 100 μg; 5 mg/mL concentration in PBS solution containing 1% bovine serum albumin (BSA) were added to a well, incubated at room temperature for 10 min, washed three times with PBS, and FRES imaging was performed for 3 min. In addition, we confirmed FRES imaging differences according to seeded cell density (0, 10^3^, 10^4^, and 10^5^ cells/well) with 10 μg of EGFR-F-SERS-A dots in the same manner.

For the *in vivo* studies, mice with CRC were initially anesthetized with an intramuscular injection of 200 µL of 0.5% zoletil 50 (tiletamine-zolazepam; Virbac S.A., Carros, France) and 0.2% xylazine (Rompun; Bayer, Leverkusen, Germany) solution (1:1). In the tumor-exposed system, after making an incision of the skin and anterior wall of the colorectal area, tumors were exposed and tumor sizes were measured using a digital caliper.

To validate the multiple targeting ability of FRES, 100 μg each of antibody-conjugated F-SERS dots (EGFR-F-SERS-A and VEGF-F-SERS-B: 20 μL volume with 5 mg/mL concentration in PBS solution containing 1% BSA) were sprayed onto the surface of a 1 × 10^7^ HT29-effluc cell-injected tumor using a micropipette (AxyPet^TM^ single-channel pipettor via the anus. After 10 min of incubation, the antibody-conjugated F-SERS dots were washed five times with 300 μL of PBS. Fluorescence and Raman spectra of tumors were simultaneously measured in the tumor area using FRES for 3 min. A single treatment with 100 μg each of antibody-conjugated F-SERS dots (EGFR-F-SERS-A or VEGF-F-SERS-B) or IgG-F-SERS-A/B dots (control) was also performed in the same manner.

To validate FRES for a real-time endoscopic system, 100 μg of antibody-conjugated F-SERS dots were sprayed onto the lumen of the colon through the anus. After 10 min of incubation, antibody-conjugated F-SERS dots-treated areas were washed five times with 300 μL of PBS. The multiplexing capability of FRES was investigated for 3 min by inserting a probe into the colon through the anus of tumor-implanted and normal mice.

To validate the lower dose limit of the antibody-conjugated F-SERS dots using FRES in a small tumor, different amounts of the former were sprayed onto the surface of a 5 × 10^6^ HT29-effluc cell-injected tumor and evaluated by FRES as described above. The groups under the study were as follows:

1. High dose: 100 μg each of EGFR-F-SERS-A and VEGF-F-SERS-B dots on a tumor.
2. Medium dose: 50 μg each of EGFR-F-SERS-A and VEGF-F-SERS-B dots on a tumor.
3. Low dose: 25 μg each of EGFR-F-SERS-A and VEGF-F-SERS-B dots on a tumor.
4. Control: 100 μg each of EGFR-F-SERS-A and VEGF-F-SERS-B dots on a normal colon.

The multiplex targeting ability of FRES was graded into three levels:

1. A “definite signal” occurred when the two known Raman signals were clearly observed after a fluorescence signal had been found [over 30 counts per second (cps) for both Raman signals], considering more than three times of background level (10 cps).
2. A “no signal” occurred when neither Raman nor fluorescence signals were observed (between 0 to 20 cps of either or both Raman signals).
3. A “probable signal” occurred when either Raman signal was between a definite and a no signal state, but not necessarily both after a fluorescence signal was found.

## Electronic supplementary material

Supplementary methods, figures and tables
