# Supplementary material for: Simultaneous Detection of EGFR and VEGF in Colorectal Cancer using Fluorescence-Raman Endoscopy
Source: Sci Rep. 2017 Apr 21;7:1035. doi: 10.1038/s41598-017-01020-y (PMC5430917; doi:10.1038/s41598-017-01020-y)
Supplement: Supplementary file 1 — Supplementary methods, figures and tables [file 41598_2017_1020_MOESM1_ESM.pdf]

(Supplements)

Simultaneous Detection of EGFR and VEGF in Colorectal Cancer using Fluorescence-Raman Endoscopy

Yong-il Kim[1-3#], Sinyoung Jeong[4#], Kyung Oh Jung[1,5], Myung Geun Song[1,5], Chul-Hee Lee[1,5], Seock-jin Chung[1,5], Ji Yong Park[1], Myeong Geun Cha[4], Sung Gun Lee[4], Bong-Hyun Jun[6], Yun-Sang Lee[1,2], Do Won Hwang[1,2], Hyewon Youn[1,3], Keon Wook Kang[1,3], Yoon-Sik Lee[7*], Dae Hong Jeong[4*] & Dong Soo Lee[1, 2*]

SUPPLEMENTARY METHODS

Chemicals and materials

Tetraethyl orthosilicate (TEOS), 3-mercaptopropyltrimethoxysilane (MPTS), ethylene glycol (EG), silver nitrate ($AgNO_3$, > 99.99%), 3-aminopropyltriethoxysilane (APTES), octylamine (OA), rhodamine B isothiocyanate (RITC), fluorescein isothiocyanate (FITC), dimethyl sulfoxide (DMSO), *N*-methyl-2-pyrrolidone (NMP), *N*-hydroxysuccinimide (NHS), *N*,*N*′-diisopropylcarbodiimide (DIC), *N*,*N*′-diisopropylethylamine (DIPEA), 4-dimethylaminopyridine (DMAP), and bovine serum albumin (BSA, > 98%) were purchased from Sigma-Aldrich Inc. (St. Louis, MO, USA) and used without further purification. Ethanol (99.9% & 98 %), ammonium hydroxide (NH_4OH, 27%), and 2-propanol (99%) were purchased from Daejung Chemicals (Siheung, Korea). Succinimidyl esters conjugated with Alexa Fluor (AF) 610-X was purchased from Invitrogen Inc. (Carlsbad, CA, USA). Phosphate-buffered saline (PBS; 137 mM NaCl, 10 mM Na_2HPO_4, 2.0 mM KH_2PO_4, and 2.7 mM KCl at pH 7.4) was prepared in-house. Deionized (DI) water was used for all experiments. Cetuximab [anti-epidermal growth factor receptor (EGFR) monoclonal antibody] and Bevacizumab [anti-vascular endothelial growth factor (VEGF) monoclonal antibody] were purchased from Merck Millipore (Darmstadt, Germany).

Immobilization of antibodies on F-SERS dots

To introduce an amino-functional group onto the surface of the F-SERS dots, 1 mg of the latter was treated with a 1 mL APTES solution (5% volume in ethanol) and 10 μL NH_4OH (27%) for 1 h at room temperature. After washing with ethanol, the amine-

functionalized F-SERS dots (1 mg) were then reacted with 1.8 mg succinic anhydride and 3 μL DIPEA in 500 μL NMP for 2 h to introduce carboxylic acid groups. Subsequently, the carboxylic acid group-functionalized F-SERS dots were activated with 2 mg NHS, 2.7 μL DIC, and 0.2 mg DMAP for 2 h. To remove excess reagents, the resulting solution was centrifuged and washed with NMP and PBS (pH 7.4). For antibody conjugation, cetuximab (50 μg) or bevacizumab (50 μg) was added either to the NHS-activated F-SERS-A or -B dots, respectively, dispersed in 200 μL of PBS. After incubation for 1 h at room temperature, the antibody-immobilized F-SERS dots were centrifuged and washed consecutively with PBS containing 0.1% (w/v) Tween 20 and then with PBS. Finally, to improve bio-compatibility, the antibody-immobilized F-SERS dots were treated with BSA [1% (w/v) in PBS solution, pH 7.4] for 30 min, washed with PBS solution containing Tween 20, and then with PBS, as described above.

Size and concentration measurements

Size distribution was measured using the nanoparticle tracking analysis (NTA) method (NanoSight NS500; Malvern, Worcestershire, UK). Samples were sufficiently diluted to minimize interference between particles. The apparatus' quick measurement mode was performed to find optimal conditions, then 5 clips of particle motion videos were recorded automatically using the standard measurement mode. Captured videos (five videos per sample) were processed and analyzed. All other conditions were constant. Graphical figures were automatically drawn by the built-in software.

Western blot analysis

Cells were lysed in radio-immunoprecipitation assay (RIPA) buffer (Sigma, St. Louis,

MO, USA) containing a protease inhibitor cocktail (Roche Diagnostics, Basel, Switzerland), and subsequently cleared via centrifugation (14,000 × g for 20 min at 4 °C). Protein concentrations were determined using BCA protein assay kits (Thermo Scientific, Rockford, IL, USA). Approximately 30 μg of protein was mixed with 4 × polyacrylamide gel electrophoresis sample buffer, boiled at 70 °C for 10 min, separated by gradient (4 – 12%) polyacrylamide gel electrophoresis, and transferred to nitrocellulose membranes (Invitrogen, Grand Island, NY, USA). Membranes were blocked with 5% skimmed milk in Tris-buffered saline (20 mM Tris, 137 mM sodium chloride, and 0.1% polysorbate 20) for 1 h at room temperature, and then incubated overnight at 4 °C with the following primary antibodies: rabbit monoclonal EGFR antibody (1:1,000 dilution; Cell Signaling, Danvers, MA, USA), rabbit polyclonal VEGF antibody (1:1,000 dilution; Abcam, Cambridge, MA, USA), and mouse monoclonal β-actin antibody (1:5,000 dilution; Sigma-Aldrich, St. Louis, MO, USA). After the membranes had been washed three times with Tris-buffered saline, they were incubated with a secondary anti-rabbit/anti-mouse horseradish peroxidase–conjugated antibody (1:2,000 dilution; Santa Cruz Biotechnology, Santa Cruz, CA, USA) for 2 h, and enhanced chemiluminescence detection reagent (Thermo Scientific, Rockford, IL, USA). Signal intensities were then measured using an LAS-3000 imaging system (Fuji Film, Tokyo, Japan).

Evaluation of the orthotopic CRC xenograft model

Mice were followed up every two days after orthotopic CRC xenograft modeling to confirm their survival. At the same time, mice were visually examined for signs of anal erosion. For bioluminescence imaging acquisition, an IVIS100 imaging system (Caliper Life Sciences, Hopkinton, MA, USA) was used. D-luciferin potassium salt was diluted to 0.3

mg/mL in PBS before use, and 100 μL of the D-luciferin solution was intraperitoneally injected into mice. The animals were anesthetized with isoflurane and transferred into a light-tight chamber equipped with a charge-coupled device camera operated by Live Image software (Xenogen Corp., Alameda, CA, USA) to obtain bioluminescence images that were serially acquired every 5 min until maximum signals were reached (time: 1 s, binning: medium, f stop: 1). Signal intensities were displayed on a pseudocolor scale. To quantify the emitted light, regions of interest (ROIs) were drawn on each bioluminescent image to quantify the optical flux (p/s/cm^2/sr: photons per second per cm^2 per steradian) over each tumor area. The same-sized ROI was drawn for comparison over the brain area, and the tumor-to-background ratio (TBR; optical flux ratio between the tumor and the brain area) was calculated. Time points for bioluminescence image acquisition were one and two weeks for the injection of 1×10^7 HT29-effluc cells groups, and one week for the injection of 5×10^6 HT29-effluc cells group.

Confocal laser scanning microscopy (CLSM)

For the *in vitro* analysis, the interaction of antibody-conjugated F-SERS dots with colon cancer cells was investigated. HT29-effluc cells (10^4 cells/well) were seeded in an 8-well chambered coverglass (Lab-Tek; Thermo Scientific, Rochester, NY, USA) with 300 μL of cell media per well. After 24 h incubation at 37 ℃, cells were fixed with 4% paraformaldehyde (Wako, Osaka, Japan) for 20 min and then washed three times with PBS. 10 µg of antibody-conjugated F-SERS dots (EGFR-F-SERS-A, VEGF-F-SERS-B, and a mixture) or BSA-F-SERS dots (control) were added to the wells, incubated at room temperature for 10 min, and then washed three times with PBS. Cell nuclei were stained by 4', 6-diamino-2-phenylindole (DAPI).

For the *in vivo* analysis, tumors were excised, fixed with 4% paraformaldehyde, and sectioned, followed by staining of the nuclei using DAPI. Confocal laser scanning microscopy (CLSM: LSM 510 META; Carl Zeiss, Jena, Germany) was used for fluorescence signal detection. Excitation laser lines for F-SERS dots and DAPI signals were 610 nm and 405 nm, respectively. Data were analyzed using LSM Image Examiner software (Carl Zeiss; Jena, Germany).

Pathologic evaluation

Immunohistochemistry (IHC) for EGFR and VEGF was carried out on formalin-fixed, paraffin-embedded serial sections cut at 3 μm and dried at 37 °C overnight. Immunostaining was performed using an avidin–biotin complex (ABC) procedure, including heat-induced epitope and enzymatic antigen retrieval procedures. The IHC for EGFR (C74B9 1:50; Cell Signaling Technologies, Beverly, MA, USA) and VEGF (VEGF-A20 1:100; Abcam, Cambridge, MA, USA) was performed according to the manufacturer's instructions. Parallel sections were stained with hematoxylin and eosin (H&E).

Statistical analysis

The correlation between protein concentration and luciferase activity in the *in vitro* study was evaluated using Spearman's correlation. Statistical significance of the *in vivo* bioluminescence studies was determined using a Wilcoxon signed rank test (TBR changes according to HT29-effluc injected cells week) and a Mann-Whitney U test (TBR difference according to HT29-effluc injected cells number). A *P*-value less than 0.05 was considered significant. All statistical analyses were performed using SPSS software (Version 18.0; SPSS Inc., Chicago, IL, USA) and MedCalc (Version 12.2; MedCalc Inc., Mariakerke, Belgium).

SUPPLEMENTARY FIGURES

Supplementary Figure 1. Characteristics of fluorescence and surface-enhanced Raman scattering nanoprobes (F-SERS dots).

Schematic (a) and transmission electron microscopy (TEM) images with high (b) and low (c) magnification of F-SERS dot silica cores, Raman active chemicals for Raman signals, and fluorescent silica shell for fluorescence signals.

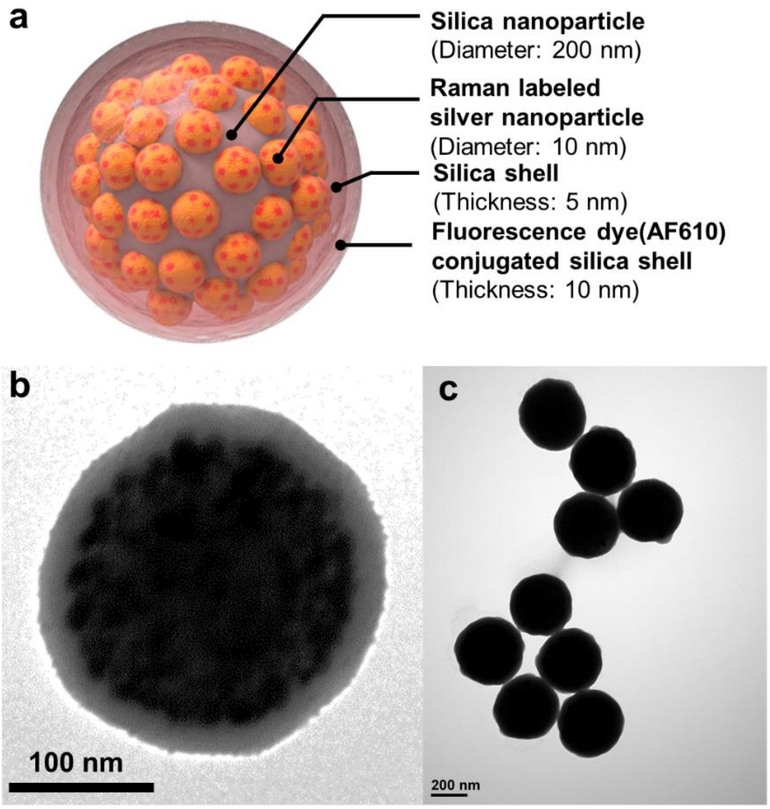

Supplementary Figure 2. Photostability of a single F-SERS dot.

The relative intensities to the initial value of (a) fluorescence (at 625 nm for AF610) and (b) SERS signals (at 1324 cm^{-1} for FITC and at 1648 cm^{-1} for RITC). The fluorescence and SERS spectra were obtained from the same single F-SERS dot with continuous laser exposure (300 s). Inset: the individual spectra of fluorescence and SERS signals obtained by 532 nm photoexcitation at 3.6 mW on the sample and 1 s acquisition time.

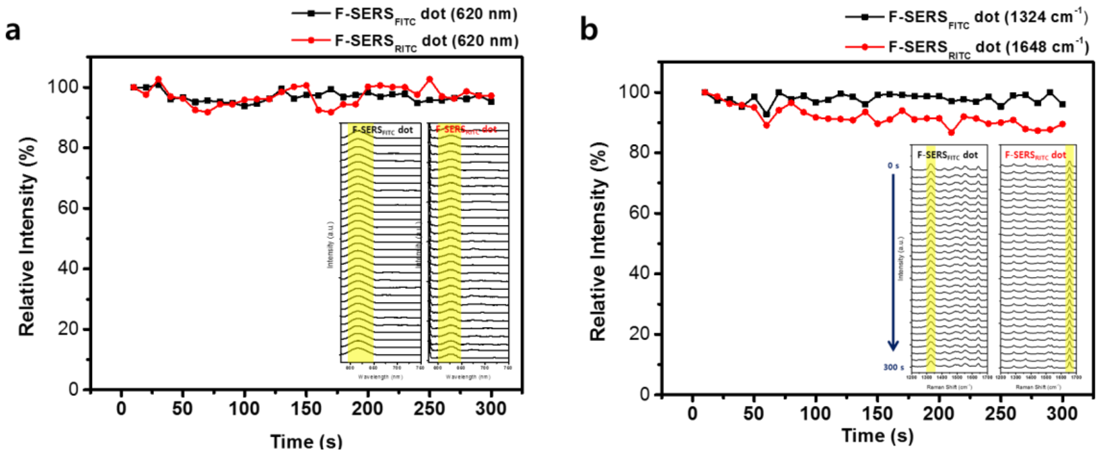

Supplementary Figure 3. *In vitro* FRES according to seeded cell density. Fluorescence signal (a) and Raman intensity at 1648 cm^{-1} (b) gradually become definite as the cell number increases.

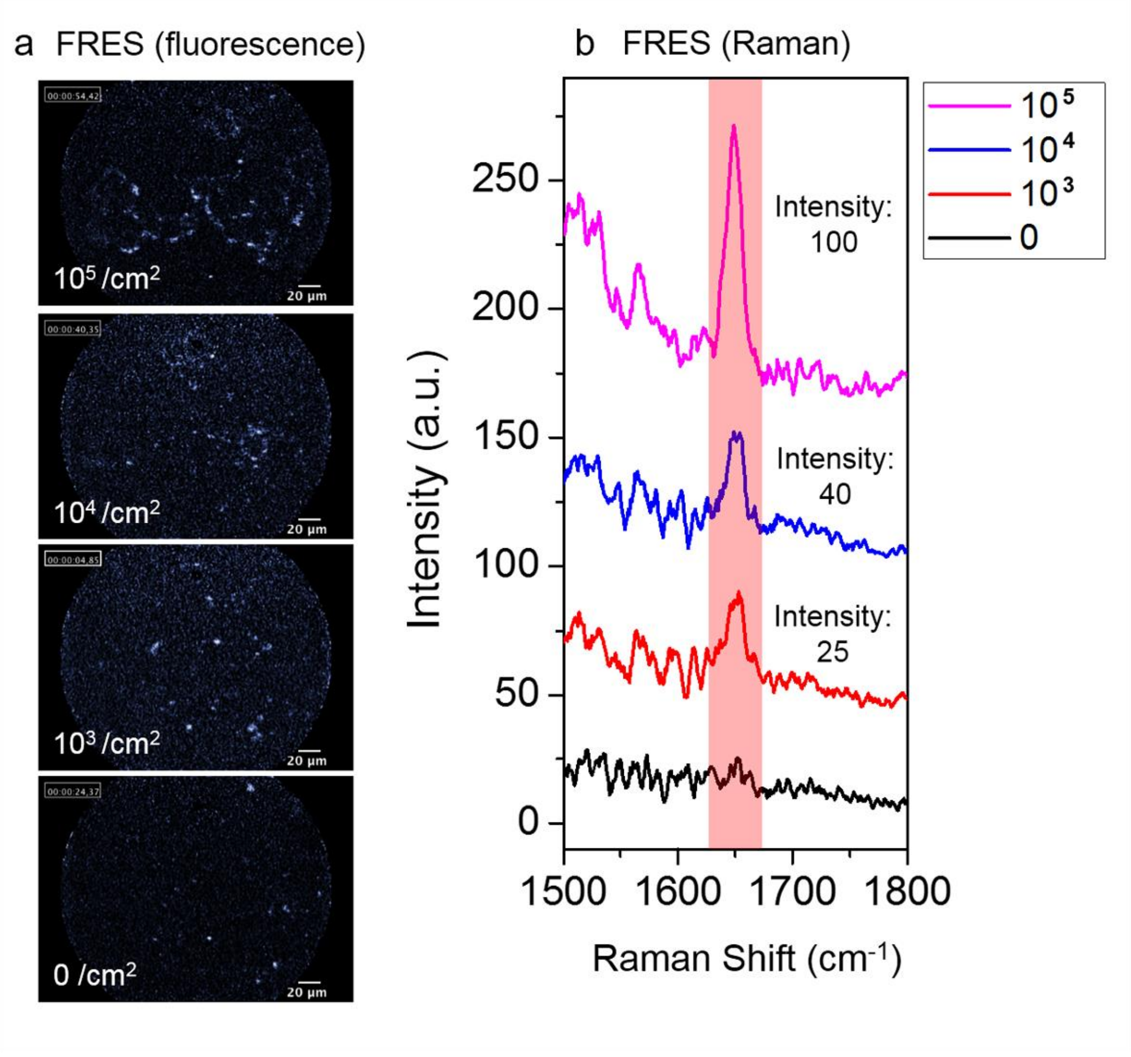

Supplementary Figure 4. Dot plot results for the validation of multiplex targeting ability. All of the multiplex or single targeting experiments showed definite or probable signals, but control (IgG-F-SERS-A/B dots) showed no signal (cps: counts per second). Mann-Whitney U test showed significant results between multiplex signals and controls (all $P = 0.036$ for Raman A and Raman B signals).

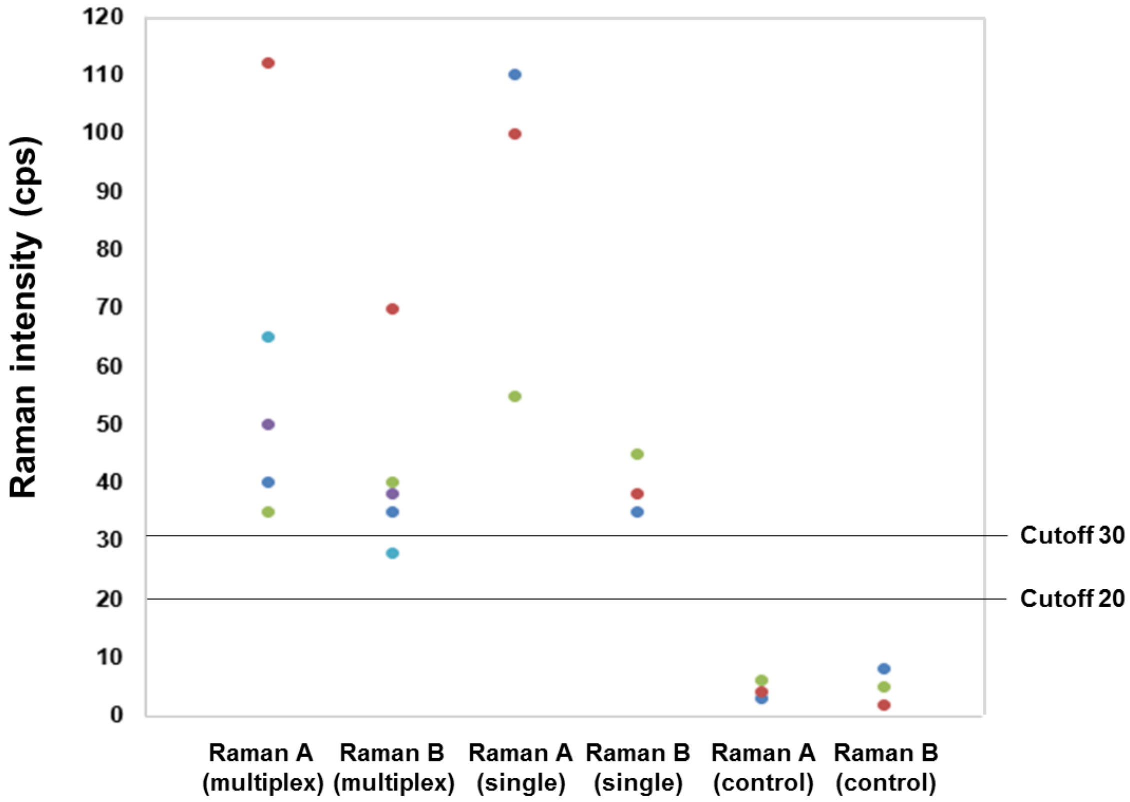

Supplementary Figure 5. CLSM for the validation of multiplex targeting ability. Tumors exposed to F-SERS dots (related to Fig. 5) were excised, fixed, and sectioned (nuclei were stained with DAPI, and CLSM was used for fluorescence signal detection). Fluorescence signals were observed for both (a) and single (b, c) antibody-conjugated F-SERS dots sprayed on tumors. No signal was found for BSA-F-SERS-A/B dots sprayed on the tumors (d).

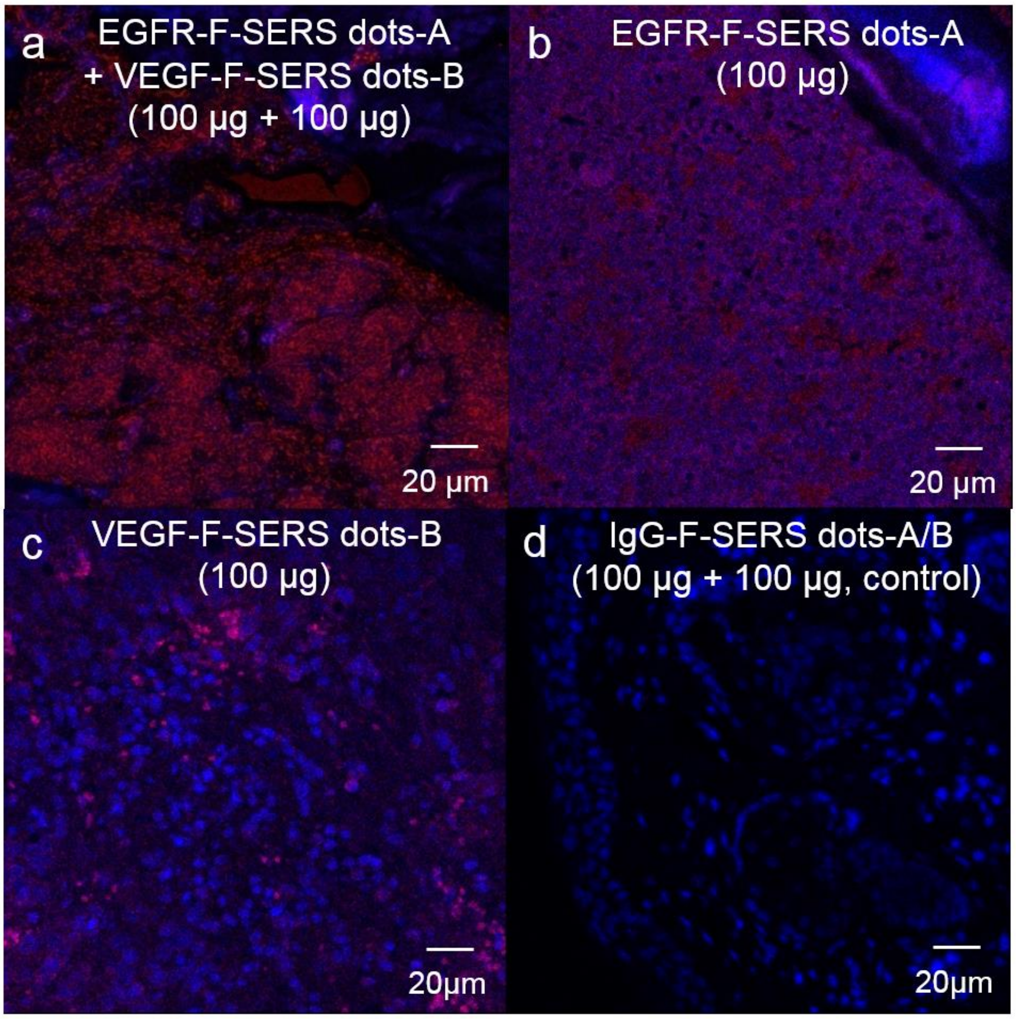

Supplementary Figure 6. Dot plot results for the real-time endoscopic system.

All of the colon cancer models showed definite or probable signals, but the normal colon study showed no signals. Mann-Whitney U test showed significant results between colon cancers and normal colons (all $P < 0.001$ for Raman A and Raman B signals).

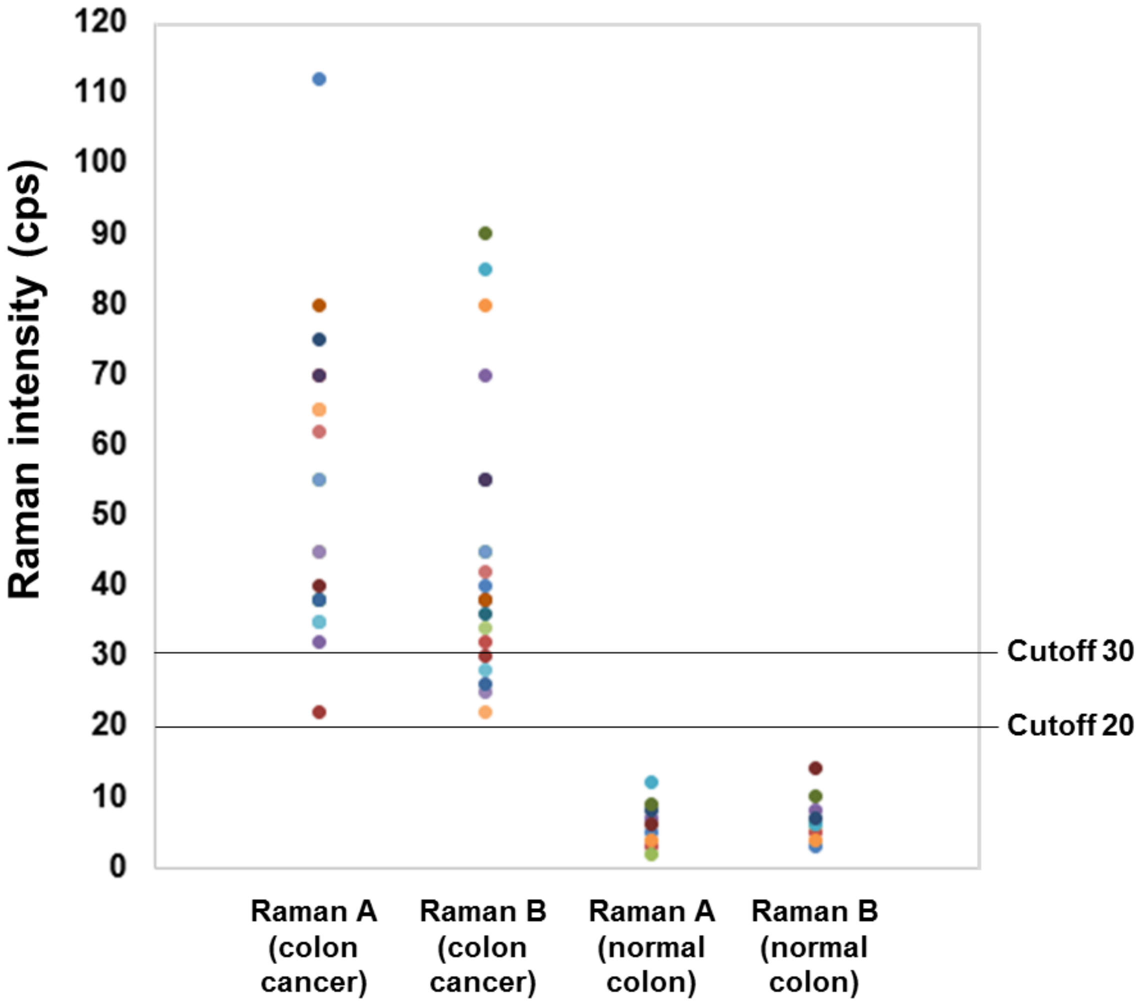

Supplementary Figure 7. Dot plot results for sensitivity and lower dose limit detection. Spraying 50 µg of antibody-conjugated F-SERS dots on colon cancer model showed definite or probable signal in all of the cases, but 25 µg of spraying antibody-conjugated F-SERS dots on colon cancer model demonstrated no signals in two cases, and the control using normal colon study showed no signals. Mann-Whitney U test showed significant results between colon cancers and normal colons (all $P < 0.001$ for each dose of Raman A and Raman B signals).

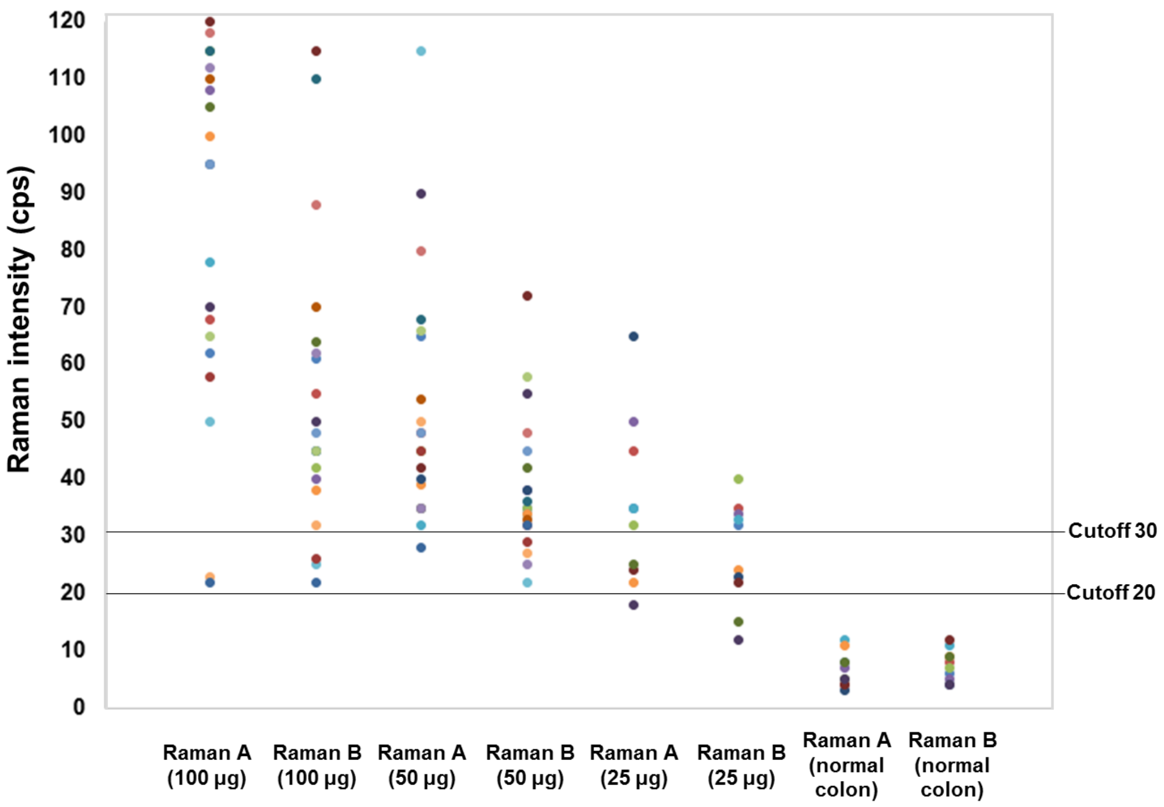

Supplementary Figure 8. CLSM for sensitivity and lower dose limit detection.

Tumors exposed to antibody-conjugated F-SERS dots (related to Fig. 6) were excised, fixed, and sectioned (nuclei were stained with DAPI, and CLSM was used for fluorescence signal detection). After spraying 100 µg (a), 50 µg (b), and 25 µg (c) of both antibody-conjugated F-SERS dots onto tumors, fluorescence signals were observed. No fluorescence signal was found after spraying 100 µg (d) of antibody-conjugated F-SERS dots onto normal colon.

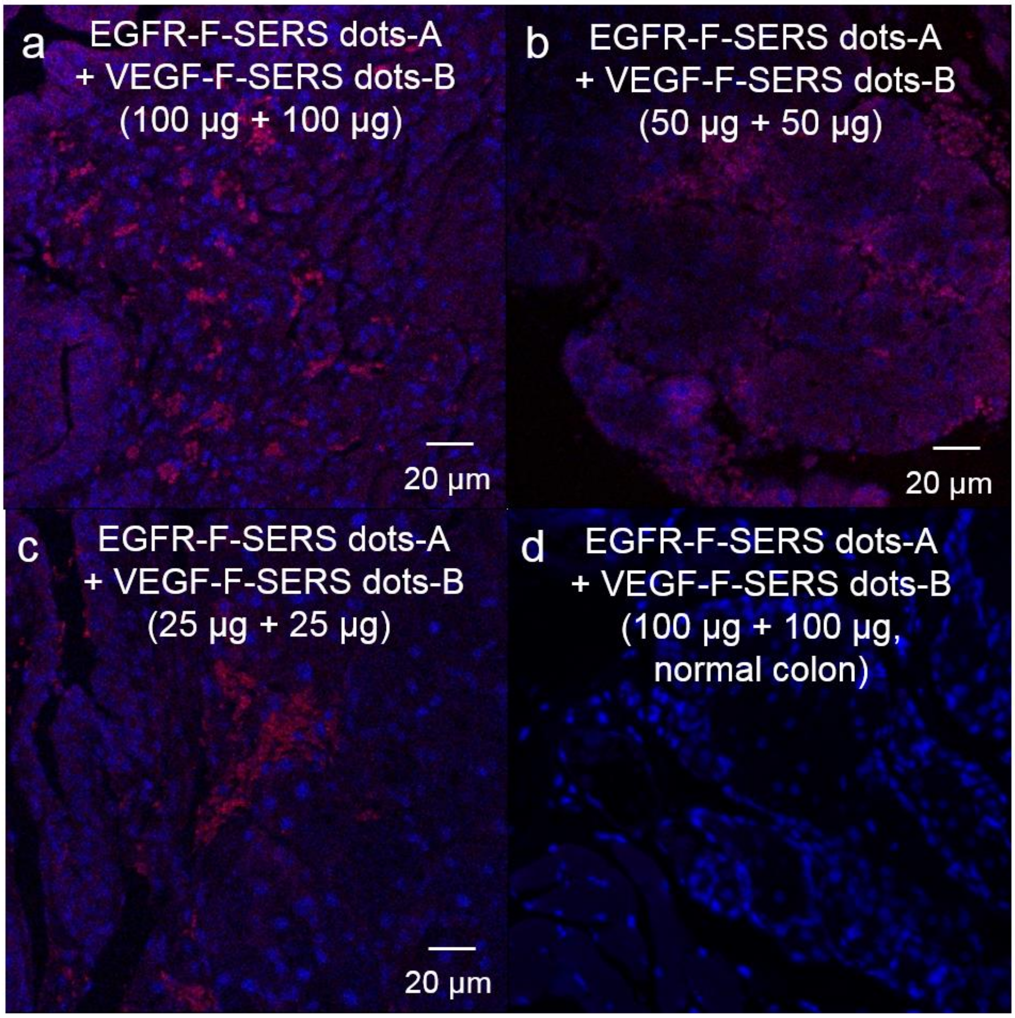

Supplementary Figure 9. Multiplex cellular targeting of tumor using FRES.

(a) Schematic illustration of the study. (b) Western blot analysis showing positive human epidermal growth factor receptor-2 (HER2) and EGFR of HT29-effluc cells. The FRES results demonstrated that mice with colon cancer (two week after injecting 1×10^7 of HT29-effluc cells) demonstrated fluorescence signals (c) and two corresponding Raman signals (d) after spraying with 100 μg each of HER2-F-SERS-A and EGFR-F-SERS-B dots.

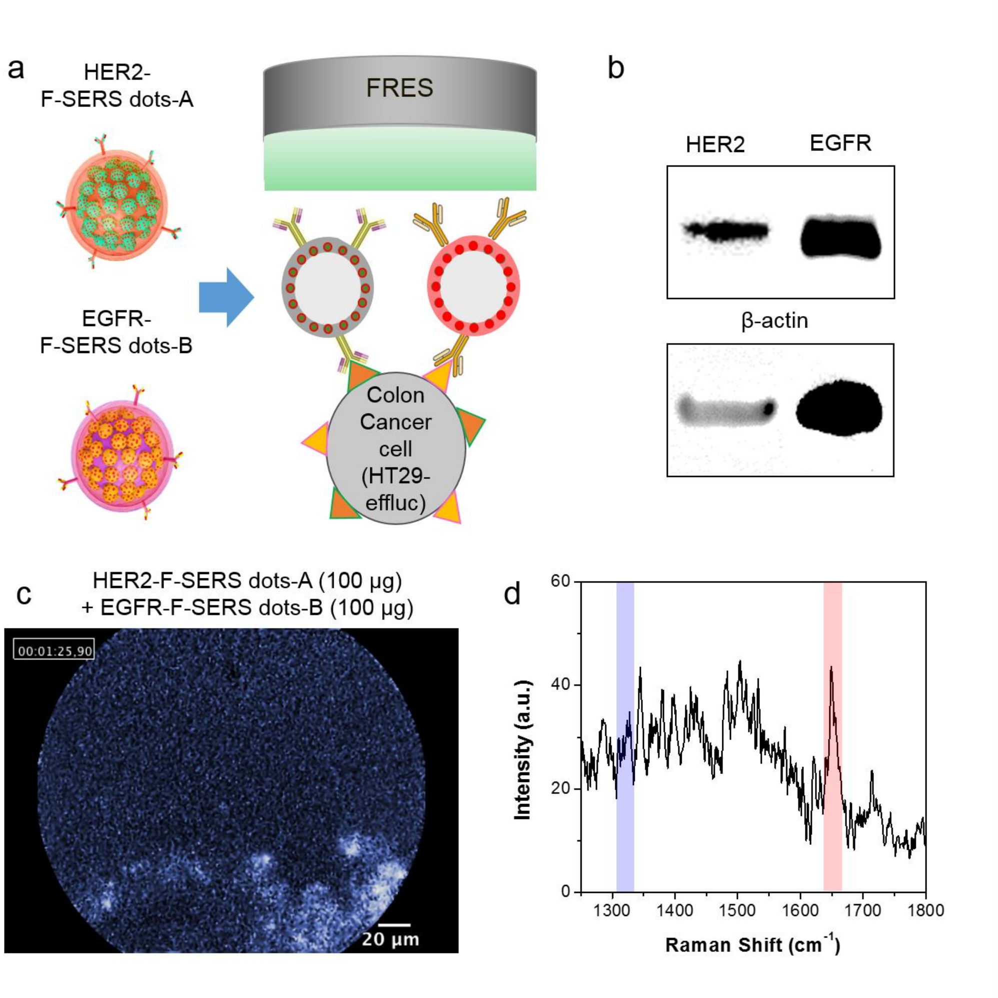

Supplementary Figure 10. Blocking study of FRES using cold antibodies.

After two weeks of injecting 1× 10^7 HT29-effluc cells orthotopically, a blocking study was carried out using cold antibodies (anti-EGFR and/or anti-VEGF) for 10 min. After blocking, 100 µg of antibody-conjugated F-SERS dots (EGFR-F-SERS-A and VEGF-F-SERS-B) were sprayed onto the tumors, which were then investigated using a FRES probe after 10 min of incubation and PBS washing. Tumors without blocking (a, b) showed definite fluorescence signals and two Raman intensities [RITC (-A) and FITC (-B)]. Single blocking using either anti-VEGF antibody (c, d) or anti-EGFR antibody (e, f) demonstrated fluorescence signals, but the Raman intensities for FITC (-B) and RITC (-A) were not found. Dual blocking by anti-EGFR and anti-VEGF antibodies (g, h) revealed no definite fluorescence or Raman signals. Dot plot results (i) demonstrated that no signal was found in all cases when using more than 50 µg of cold antibodies, however, some probable signals were found when using 25 µg of cold antibodies. Immunohistochemistry (IHC) results showed positivity for EGFR (j) and VEGF (k) in colon cancer. In contrast, negative results were found in normal colon.

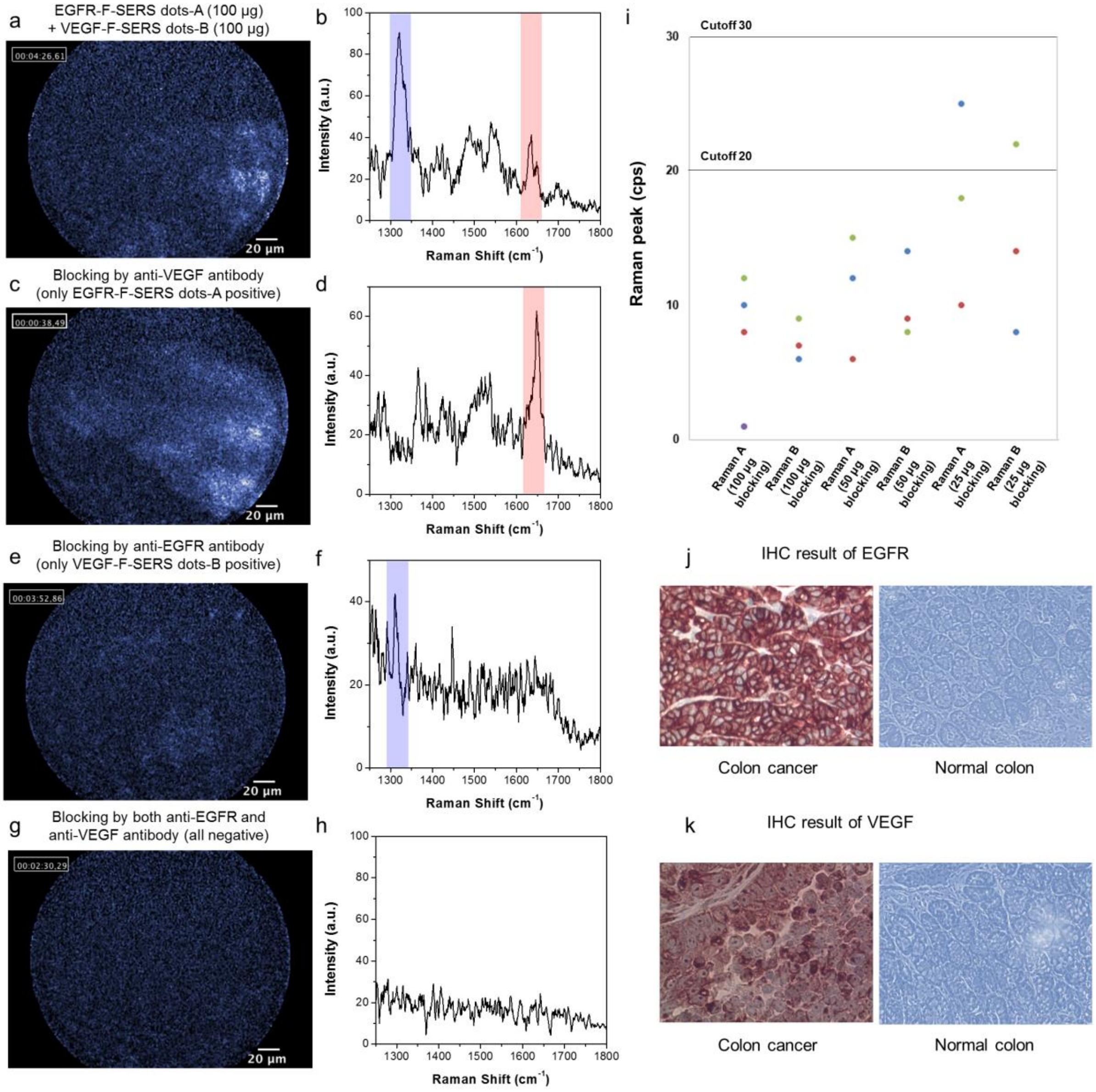

a
EGFR-F-SERS dots-A (100 µg) + VEGF-F-SERS dots-B (100 µg)
20 µm
b
Intensity (a.u.)
Raman Shift (cm-1)
c
Blocking by anti-VEGF antibody (only EGFR-F-SERS dots-A positive)
d
e
Blocking by anti-EGFR antibody (only VEGF-F-SERS dots-B positive)
f
g
Blocking by both anti-EGFR and anti-VEGF antibody (all negative)
h
i
Cutoff 30
Cutoff 20
Raman peak (cps)
Raman A (100 µg blocking)
Raman B (100 µg blocking)
Raman A (50 µg blocking)
Raman B (50 µg blocking)
Raman A (25 µg blocking)
Raman B (25 µg blocking)
j
IHC result of EGFR
Colon cancer
Normal colon
k
IHC result of VEGF
Colon cancer
Normal colon

Supplementary Figure 11. Detection of heterogeneous EGFR expression by FRES *ex vivo.* One week after subcutaneous injection of 1× 10^7 HT29-effluc cells, tumors were excised and divided into halves. 100 μg of EGFR-F-SERS-A dots were sprayed onto the tumors and tissues were investigated using FRES. Tumors expressing definite fluorescence (a) and Raman intensity (b) showed intense EGFR expressions with IHC (c). On the contrary, tumors showing probable fluorescence (d) and Raman intensity (e) demonstrated moderate EGFR expression with IHC (f).

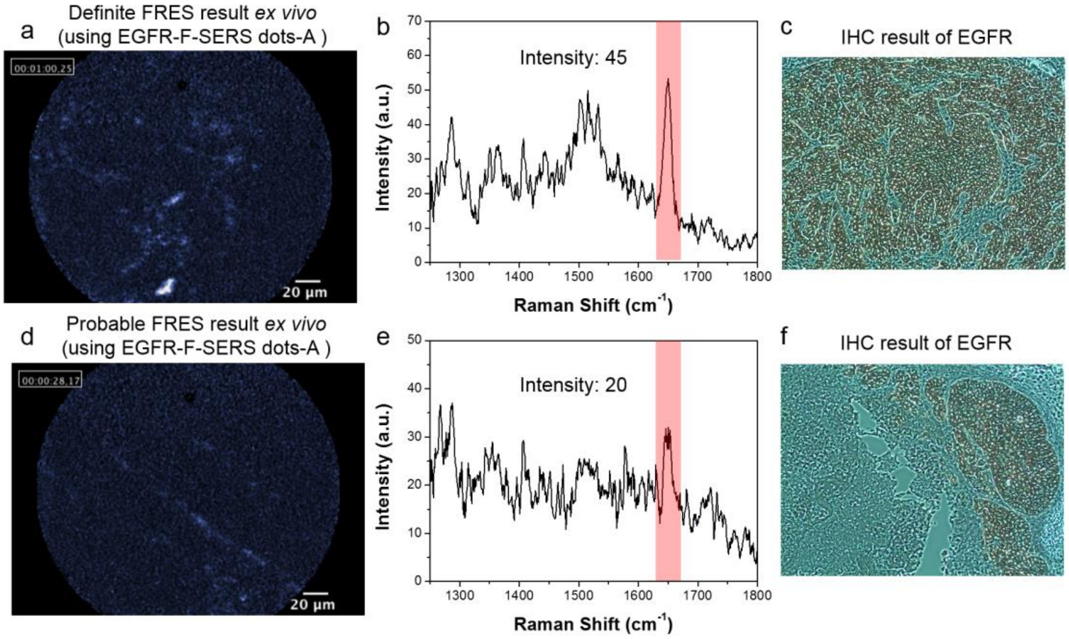

Supplementary Figure 12. Description of devices and optical results for FRES.

(a) A schematic diagram of the optical beam path (Mx: oscillating mirror for the X-axis, My: oscillating mirror for the Y-axis, BS: beam splitter, DF: dichroic filter, and LP: long pass Raman edge filter). (b) An illustration of the spectrum of collected lights from FRES. The collected lights contained four different kinds of signals: 1) scattered laser-line (Rayleigh scattering), 2) a Raman scattering signal due to intrinsic bare fiber, 3) fluorescence, and 4) SERS signals from F-SERS dots. (c) Photograph of the FRES apparatus.

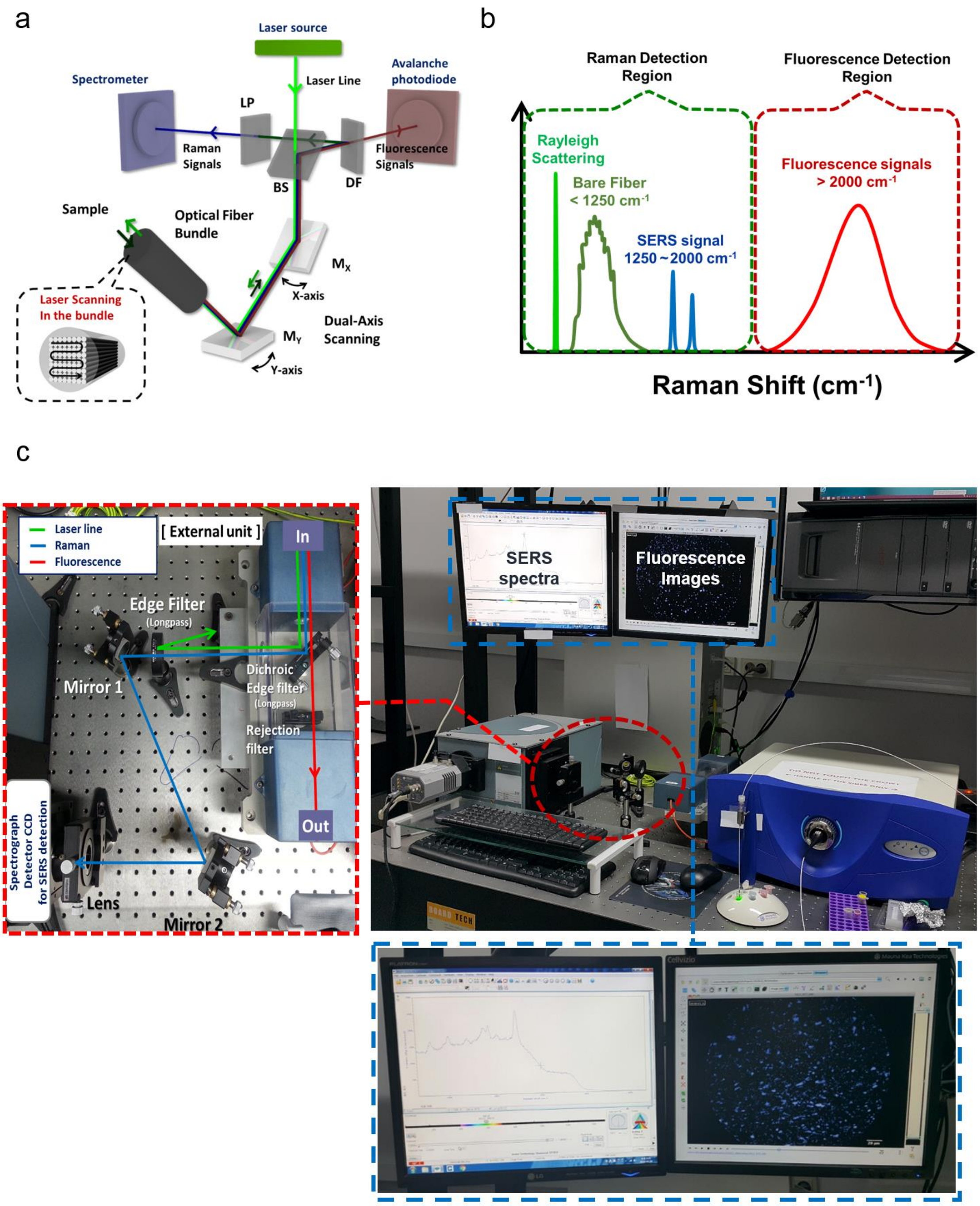
a
Laser source
Laser Line
Spectrometer
Avalanche photodiode
LP
Raman Signals
Fluorescence Signals
BS
DF
Sample
Optical Fiber Bundle
Mx
X-axis
Laser Scanning In the bundle
My
Y-axis
Dual-Axis Scanning
b
Raman Detection Region
Fluorescence Detection Region
Rayleigh Scattering
Bare Fiber
< 1250 cm-1
SERS signal
1250~2000 cm-1
Fluorescence signals
> 2000 cm-1
Raman Shift (cm-1)
c
Laser line
Raman
Fluorescence
[External unit]
In
Out
Edge Filter
(Longpass)
Mirror 1
Dichroic Edge filter
(Longpass)
Rejection filter
Spectrograph Detector CCD for SERS detection
Lens
Mirror 2
SERS spectra
Fluorescence Images

Supplementary Figure 13. Full-length western blot analysis results of EGFR and VEGF.

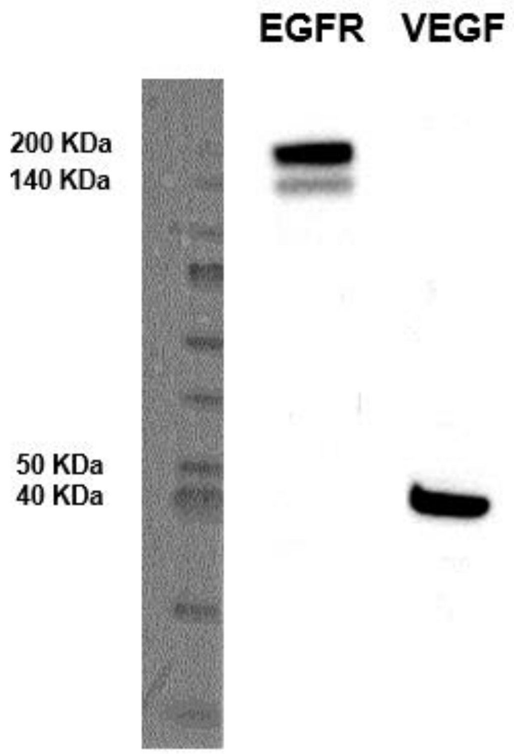

SUPPLEMENTARY TABLES

Supplementary Table 1. Summary of dynamic light scattering (DLS) and nanoparticle tracking analysis (NTA) results for F-SERS dots and antibody-conjugated F-SERS dots

agents	mean size (nm)	standard deviation (SD, nm)	polydispersity index (PDI)	zeta potential (mV)
F-SERS dots-A	318.6	20.1	0.207	-21
EGFR-F-SERS dots-A	352.5	37.1	0.171	-14
IgG-F-SERS dots-A	359.3	22.6	0.159	-11
F-SERS dots-B	326.3	25.9	0.192	-25
VEGF-F-SERS dots-B	363.8	34.3	0.155	-10
IgG-F-SERS dots-B	367.5	28.8	0.178	-17

F-SERS = fluorescence and surface-enhanced Raman scattering; EGFR = epidermal growth factor receptor; VEGF = vascular endothelial growth factor

Supplementary Table 2. Clinical results of colorectal cancer orthotopic xenograft modeling

time after 1×10^7 HT29-effluc cells injection	survival	anal erosion
1 week	100% (20/20)	35% (7/20)
2 week	70% (14/20)	86% (12/14)

Supplementary Table 3. Multiplex targeting ability validation of FRES

spraying materials	definite signal	probable signal	no signal
EGFR-F-SERS dots-A + VEGF-F-SERS dots-B (100 µg + 100 µg, multiplex targeting)	80% (4/5)	20% (1/5)	0% (0/5)
EGFR-F-SERS dots-A (100 µg, single targeting)	100% (3/3)	0% (0/3)	0% (0/3)
VEGF-F-SERS dots-B (100 µg, single targeting)	100% (3/3)	0% (0/3)	0% (0/3)
BSA-F-SERS dots-A/B (100 µg + 100 µg, control)	0% (0/3)	0% (0/3)	100% (3/3)

FRES = fluorescence-Raman endoscopic system

Supplementary Table 4. Confirmation of the usefulness of FRES in a real-time endoscopic system

spraying dose (EGFR-F-SERS dots-A + VEGF-F-SERS dots-B)	definite signal	probable signal	no signal
100 µg + 100 µg	75% (15/20)	25% (5/20)	0% (0/20)
100 µg + 100 µg (normal colon; control)	0% (0/9)	0% (0/9)	100% (9/9)

Supplementary Table 5. Sensitivity and lower limit dose identification of FRES

spraying dose (EGFR-F-SERS dots-A + VEGF-F-SERS dots-B)	definite signal	probable signal	no signal
100 µg + 100 µg	80% (16/20)	20% (4/20)	0% (0/20)
50 µg + 50 µg	75% (15/20)	25% (5/20)	0% (0/20)
25 µg + 25 µg	50% (5/10)	30% (3/10)	20% (2/10)
100 µg + 100 µg (normal colon; control)	0% (0/8)	0% (0/8)	100% (8/8)
